# Evidence of innate training in bovine γδ T cells following subcutaneous BCG administration

**DOI:** 10.3389/fimmu.2024.1423843

**Published:** 2024-07-18

**Authors:** Beulah Esther Rani Samuel, Fabian E. Diaz, Teresia W. Maina, Ryan J. Corbett, Christopher K. Tuggle, Jodi L. McGill

**Affiliations:** ^1^ Department of Veterinary Microbiology and Preventive Medicine, Iowa State University, Ames, IA, United States; ^2^ Immunology, Cargill Animal Nutrition & Health, Elk River, MN, United States; ^3^ Center for Data Driven Discovery, Children’s Hospital of Philadelphia, Philadelphia, PA, United States; ^4^ Department of Animal Science, Iowa State University, Ames, IA, United States

**Keywords:** trained immunity, bovine γδ T cells, chromatin accessibility, BCG vaccine, epigenetic reprogramming

## Abstract

The Bacillus Calmette Guerin (BCG) vaccine has been shown to induce non-specific protection against diseases other than tuberculosis in vaccinated individuals, attributed to the induction of trained immunity. We have previously demonstrated that BCG administration induces innate immune training in mixed peripheral blood mononuclear cells and monocytes in calves. Gamma Delta (γδ) T cells are non-conventional T cells that exhibit innate and adaptive immune system features. They are in higher proportion in the peripheral blood of cattle than humans or rodents and play an essential role in bovine immune response to pathogens. In the current study, we determined if BCG administration induced innate immune training in bovine γδ T cells. A group of 16 pre-weaned Holstein calves (2-4 d age) were enrolled in the study and randomly assigned to vaccine and control groups (n=8/group). The vaccine group received two doses of 10^6^ colony forming units (CFU) BCG Danish strain subcutaneously, separated by 2 weeks. The control group remained unvaccinated. Gamma delta T cells were purified from peripheral blood using magnetic cell sorting three weeks after receiving the 1^st^ BCG dose. We observed functional changes in the γδ T cells from BCG-treated calves shown by increased IL-6 and TNF-α cytokine production in response to *in vitro* stimulation with *Escherichia coli* LPS and PAM3CSK4. ATAC-Seq analysis of 78,278 regions of open chromatin (peaks) revealed that γδ T cells from BCG-treated calves had an altered epigenetic status compared to cells from the control calves. Differentially accessible peaks (DAP) found near the promoters of innate immunity-related genes like *Siglec14*, *Irf4*, *Ifna2*, *Lrrfip1*, and *Tnfrsf10d* were 1 to 4-fold more accessible in cells from BCG-treated calves. MOTIF enrichment analysis of the sequences within DAPs, which explores transcription factor binding motifs (TFBM) upstream of regulatory elements, revealed TFBM for Eomes and IRF-5 were among the most enriched transcription factors. GO enrichment analysis of genes proximal to the DAPs showed enrichment of pathways such as regulation of IL-2 production, T-cell receptor signaling pathway, and other immune regulatory pathways. In conclusion, our study shows that subcutaneous BCG administration in pre-weaned calves can induce innate immune memory in the form of trained immunity in γδ T cells. This memory is associated with increased chromatin accessibility of innate immune response-related genes, thereby inducing a functional trained immune response evidenced by increased IL-6 and TNF-α cytokine production.

## 1 Introduction

Epidemiological studies have shown that vaccination with Bacillus Calmette–Guérin (BCG) vaccine can induce non-specific protection against diseases other than tuberculosis, reducing all-cause mortality in humans (1, 2). This non-specific protection is partially attributed to the induction of trained immunity, which acts through epigenetic reprogramming, influencing the activation of innate receptors and the release of cytokine mediators (3, 4). The BCG vaccine has been well studied in cattle to prevent bovine tuberculosis (5, 6), stimulating interest in exploring BCG-induced trained immunity in cattle. Previous studies from our lab group have reported that BCG administration can induce trained immunity in peripheral blood mononuclear cells (PBMCs) (7) and monocytes (8) in neonatal calves.

Trained immunity has been demonstrated in many cell types in humans and rodents, such as monocytes, NK cells, neutrophils, and epithelial cells (9–12). Gamma delta (γδ) T cells are unique leukocytes showing diverse innate and adaptive characteristics, and studies have demonstrated their role in vaccine-mediated protection against infectious diseases in humans, including BCG-immunized infants (13, 14). γδ T cells are known to recognize non-peptide antigens without MHC restriction, making them distinct from αβ T cells (15, 16). Recent studies have observed that trained immunity can be induced in human γδ T cells by administration of live attenuated vaccines such as the MMR vaccine (17) and the BCG vaccine (18). Cattle are a “γδ high” species, with increased frequencies of circulating γδ T cells compared to humans and rodents (19). In young ruminants especially, which lack serum protective antibodies at birth and depend on colostrum, γδ T cells are found at increased frequencies (up to 50% of the PBMCs) (19) and play a significant role in protection till αβ T cell memory is established (20).

Hedges et al. (21) demonstrated that purified human and bovine γδ T cells are more like myeloid cells rather than the αβ T cells in their ability to respond to PAMPs through innate receptors in the absence of antigen-presenting cells. Human γδ T cells were observed to express several cytokines and chemokines such as TNF-α, IFN-γ, GM-CSF, CCL4, and MIP-1α following LPS stimulation. Bovine γδ T cells, in addition to the cytokines and chemokines, express pathogen recognition receptors (TLR 1, TLR 2, TLR 3, TLR 4, TLR 7, TLR 8, and TLR 9), adaptor proteins (MyD88, TRAM, SARM), CD14, CD11b, the glycoprotein CD38, and scavenger receptors (21). Serrano et al. (22) reported γδ T cells can be activated by monocyte-dependent co-stimulation with TLR 8 ligands in mice. Mokuno et al. (23) demonstrated the expression of TLR 2 in mice Vγ6/Vδ1 T cells and their involvement in the activation of these cells by *E. coli* lipid A. Meissner et al. (24) detected myeloid surface receptors such as CD14, CD68, and scavenger receptor-1 and secreted molecules such as TNF, G-CSF, and IL-1β on characterization of gene expression profile of circulating bovine γδ T cells stimulated with PMA/Ionomycin. Ruminant γδ T cells also uniquely express a transmembrane glycoprotein, workshop cluster 1 (WC1), that acts as a co-receptor for the γδ TCR and can bind to pathogenic surfaces as a pathogen recognition receptor (25). In ruminants, γδ T cells respond to bacterial, viral, fungal, and protozoan infections with local and systemic immune responses (26, 27). In addition, WC1 + γδ T cells are known to respond to BCG vaccination in cattle (28); however, the impact of BCG administration on γδ T cells and their capacity for mounting a trained immune response has not been explored in cattle.

The underlying molecular basis of trained immunity has been attributed to genome-wide epigenetic reprogramming of innate immune cells influenced by multiple factors like chromatin modification and organization at the level of topologically associated domains (TADs), transcription of long non-coding RNAs, DNA methylation and reprogramming of cellular metabolism (29). Specifically, studies on the mechanism of BCG-induced trained immunity have described the role of histone marks responsible for its heterogeneous effects. Increased trimethylation of histone H3 at lysine K4 (H3K4me3), associated with increased transcription of proinflammatory cytokine genes, was observed in human monocytes following BCG administration (30). Histone methylation (H3K4me3) has also been reported to play a role in BCG-induced innate immune training in mature neutrophils (12). Increased H3K27Ac signal linked to functional reprogramming and protection to non-related viral infections was observed in monocytes in BCG-immunized individuals (31).

Studies on the contribution of chromatin modification to the regulation of innate immunity have observed that proinflammatory gene loci are repressed in the quiescent cells by restricted chromatin access, and stimulation of these cells leaves an ‘epigenetic scar’ at the stimulated gene leading to long-term functional responsiveness (29, 32). Epigenetic reprogramming through histone modifications increases transcription of innate immune genes by increasing chromatin accessibility in those regions (33). Assay of transposase accessible chromatin sequencing (ATAC-seq) is designed to study chromatin accessibility and has been applied to depict enhancer landscapes and evaluate accessibility changes between normal and diseased states (34–36). In mouse hematopoietic stem cells, ATAC-seq analysis revealed that BCG exposure increases chromatin accessibility, increasing binding sites for the interferon regulatory factor, STAT1, and STAT2 family members (37). Similarly, in their ATAC seq data, Steven et al. (38) found that BCG immunization increased the accessibility of genes related to specific immune and metabolic pathways.

Knowing the innate functions of γδ T cells and their potential to recognize and respond to BCG, our objective was to determine if BCG administration induced trained immunity in γδ T cells from neonatal calves. In this study, we observed a trained phenotype in γδ T cells from calves following *in vivo* BCG vaccination, defined by enhanced production of proinflammatory cytokines in response to heterologous stimulus. We further evaluated the altered epigenetic status in γδ T cells from BCG-immunized calves compared to unvaccinated controls through differential chromatin accessibility and identified potential key regulators of the trained immune phenotype.

## 2 Materials and methods

### 2.1 Animal care

The samples used in this study were obtained from a more extensive study comprising the rBCG-N-hRSV vaccine group, wild-type BCG vaccine group, and an unvaccinated control group; the results have been published previously (39). We used samples from the wild-type BCG and control calves in this trial. A group of 16 two to four-day-old, colostrum-replete male Holstein calves were enrolled in the study. The animals were housed in a climate-controlled BSL-2 environment in the livestock infectious disease isolation facility (LIDIF) at Iowa State University. The calves were fed with a milk replacer, and water was provided ad libitum. A veterinarian supervised the animals throughout the study. All procedures in the study followed the Institutional Animal Care and Use Committee (IACUC-18-232) and Institutional Biosafety Committee (IBC-18-076) guidelines.

### 2.2 BCG immunization

Calves were acclimatized for five days from the day of arrival. The calves were separated into two groups of eight each (vaccine group n=8, control group n=8). The vaccine group received 10^6^ CFU BCG in 500 μL of sterile saline subcutaneously in the right neck. Two weeks after primary vaccination, a booster of 10^6^ CFU BCG was administered to the right neck. The control group remained unvaccinated. The vaccine group was monitored for changes in body temperature and injection site reaction after administration of BCG.

### 2.3 PBMC isolation, cryopreservation, and γδ T cell separation

Peripheral blood was collected one week after the second dose of BCG by jugular venipuncture. As described previously, PBMCs were separated from peripheral blood by density gradient centrifugation (39). In brief, peripheral blood was diluted in a 1:1 ratio with sterile PBS and centrifuged to get the buffy coat. The buffy coat layer was collected and centrifuged over Histopaque-1077 (Sigma-Aldrich) to separate the PBMCs. The contaminating RBCs were removed by hypotonic lysis. The cells were then washed in sterile PBS and counted.

As previously described, Magnetic-activated cell sorting (MACS) was used to separate γδ T cells from the PBMCs (40). The PBMCs were labeled with mouse anti-bovine γδ T cell receptor clone GB21A (Washington State Monoclonal Antibody Center, Pullman, WA) at 10 μg/mL concentration. Then, the cells were washed, resuspended in MACS buffer (0.5% BSA, 2 mM EDTA in PBS), and labeled with anti-mouse IgG2a+b microbeads (Miltenyi Biotec). The cells were incubated at 4°C for 15 mins and positively selected by passing through a magnetic column, washed, and resuspended in complete RPMI consisting of RPMI-1640 (Gibco, Carlsbad, CA) supplemented with 2 mM L-glutamine, 1% antibiotic-antimycotic solution, 1% non-essential amino acids, 2% essential amino acids, 1% sodium pyruvate, 50 μM 2-mercaptoethanol (all from Sigma, St. Louis, MO), and 10% (v/v) fetal bovine serum (FBS), and enumerated on the automated cell counter (Countess II FL automated cell counter, Thermo Fisher Scientific). The purity of γδ T cells were analyzed by flow cytometry and found to be >95% in all samples.

PBMCs were isolated two weeks after the booster and cryopreserved as previously described for future ATAC sequencing (7). Briefly, 2 x 10^7^ PBMCs were resuspended in 1 mL of pre-cooled FBS containing 10% dimethyl sulfoxide (DMSO) (Sigma-Aldrich). The cells were transferred to cryovials and moved to a -80°C freezer in polystyrene containers for a slow drop in temperature. The cryovials were transferred to a liquid nitrogen tank after 24 hours, where they remained until further processing.

### 2.4 *In vitro* stimulation and ELISA

γδ T cells were plated at a concentration of 10^6^ cells/mL. The cells were stimulated with either cRMPI only, *E. coli* lipopolysaccharide (#tlrl-b5lps, Invivogen) at a concentration of 1μg/ml in cRPMI, or PAM3CSK4 (#tlrl-pms, Invivogen) at a concentration of 10 μg/ml in cRPMI for 72 hours at 37°C. The supernatants were collected by pelleting cells at 470 x g for 5 mins and stored at -80°C until ELISA was performed. The supernatants were diluted 1:2 with the reagent diluent (4% BSA in phosphate buffered saline) and TNF-α and IL-6 ELISA were performed using commercial kits (Bovine TNF-α ELISA and Bovine IL-6 ELISA, Kingfisher Biotech). The assays were performed following the manufacturer’s instructions.

### 2.5 ATAC-Seq DNA library generation and sequencing

Cryopreserved PBMCs were removed from the liquid nitrogen tank and thawed at 37°C in a water bath for 2 mins. The cells were transferred immediately to a 15mL conical centrifuge tube containing 9 ml of warm cRPMI to remove DMSO. The cells were washed twice and counted. The cell viability was >85%. As explained above, magnetic-activated cell sorting was used to separate γδ T cells from the PBMCs.

Generation of DNA libraries for ATAC-seq was performed following the protocol by (41) with modification to the lysis step and transposition buffer incorporated from the Omni-ATAC protocol (42). One million cells were lysed, and up to 100,000 nuclei were used in the transposition reaction using the Illumina Tagment DNA Enzyme and Buffer kit (#20034197, Illumina, Inc.). The reagent volumes were adjusted according to the number of nuclei used. DNA libraries were generated using the Ad1_noMX primer and indexing primers Ad2.1 to Ad2.12. Information on the volume of reagents used and the number of cycles is provided in **Supplementary Material 1**. The indexing primers used in PCR amplification are listed in **Table 1**. The unique sequences of the primers which help in distinguishing samples in pooled libraries are in bold. The libraries were purified using AMPure XP beads (Beckman Coulter) by double-sided bead purification. The quality of libraries was analyzed in the 4150 TapeStation system (Agilent Technologies), and the libraries were stored at -20°C till sequencing. The DNA traces obtained from TapeStation are available in **Supplementary Materials 2**, **3**. The libraries were pooled in equimolar amounts to a final concentration of 1.5nM in 150μL and sequenced on Novaseq6000 (Illumina, Inc.) using an entire SP flow cell to generate 2x100 paired end reads at the Iowa State University DNA facility.

**Table 1:** PCR primers for ATAC-seq DNA library preparation.

Primer	Sequence
Ad1_noMX	5’-AATGATACGGCGACCACCGAGAT**CTACACTC**GTCGGCAGCGTCAGATGTG-3’
Ad2.1_TAAGGCGA	CAAGCAGAAGACGGCATACGAGAT**TCGCCTTA**GTCTCGTGGGCTCGGAGATGT
Ad2.2_CGTACTAG	CAAGCAGAAGACGGCATACGAGAT**CTAGTACG**GTCTCGTGGGCTCGGAGATGT
Ad2.3_AGGCAGAA	CAAGCAGAAGACGGCATACGAGAT**TTCTGCCT**GTCTCGTGGGCTCGGAGATGT
Ad2.4_TCCTGAGC	CAAGCAGAAGACGGCATACGAGAT**GCTCAGGA**GTCTCGTGGGCTCGGAGATGT
Ad2.5_GGACTCCT	CAAGCAGAAGACGGCATACGAGAT**AGGAGTCC**GTCTCGTGGGCTCGGAGATGT
Ad2.6_TAGGCATG	CAAGCAGAAGACGGCATACGAGAT**CATGCCTA**GTCTCGTGGGCTCGGAGATGT
Ad2.7_CTCTCTAC	CAAGCAGAAGACGGCATACGAGAT**GTAGAGAG**GTCTCGTGGGCTCGGAGATGT
Ad2.8_CAGAGAGG	CAAGCAGAAGACGGCATACGAGAT**CCTCTCTG**GTCTCGTGGGCTCGGAGATGT
Ad2.9_GCTACGCT	CAAGCAGAAGACGGCATACGAGAT**AGCGTAGC**GTCTCGTGGGCTCGGAGATGT
Ad2.10_CGAGGCTG	CAAGCAGAAGACGGCATACGAGAT**CAGCCTCG**GTCTCGTGGGCTCGGAGATGT
Ad2.11_AAGAGGCA	CAAGCAGAAGACGGCATACGAGAT**TGCCTCTT**GTCTCGTGGGCTCGGAGATGT
Ad2.12_GTAGAGGA	CAAGCAGAAGACGGCATACGAGAT**TCCTCTAC**GTCTCGTGGGCTCGGAGATGT

The unique sequences of the primers which help in distinguishing samples in pooled libraries are in bold.

### 2.6 ATAC-Seq data analysis

The DNA libraries were sequenced at a depth greater than 50 million reads per sample. The sequencing quality was assessed using FastQC (43), and the sequencing reads were trimmed of Illumina adapter sequences and low-quality bases using Trim Galore! (44). The trimmed reads were aligned to the bovine reference genome (ARS-UCD1.3) using BWA (45), and reads with low-quality scores mapped to the mitochondrial genome and multi-mapped reads were removed. Removal of duplicate reads was performed, and the clean reads were used for peak calling. Peak calling was done using MACS2 software (46), and each library’s Fraction of reads in peak (FRiP) score was calculated. Differential accessibility analysis was carried out using edgeR (47), and the differentially accessible peaks (DAPs) were annotated to known genomic features using the ChIPSeeker R package (48, 49). The DAP results can be accessed in **Supplementary Material 4**. Gene Ontology (GO) enrichment analysis of genes with a DAP in a promoter, intronic, or proximal intragenic region (within 5kb of gene transcription start site) was performed using the Panther database (50, 51). Transcription factor binding motif (TFBM) discovery and TFBM enrichment analysis of DAPs were carried out using the MEME suite of tools (52, 53). GO enrichment and TFBM enrichment results are available in **Supplementary Materials 5**, **6**.

### 2.7 Quantitative PCR verification of genes related to DAPs

To integrate chromatin accessibility results with gene expression, a few genes were selected from the DAP gene list by filtering genes with <0.1 false discovery rate, distance to the transcription start site within 10kb, fold change >0, and genes with immune-related functions. *In vitro* stimulation, RNA extraction, and quantitative PCR were performed as previously described (7). γδ T cells from the BCG-treated and control calves were plated at a concentration of 10^6^ cells/mL and stimulated *in vitro* with *E. coli* Lipopolysaccharide (1 μg/mL) or PAM3CSK4 (10 μg/mL) or PolyIC (50μg/ml) and Imiquimod (10μg/ml) at 37°C for 4 hours. The plates were centrifuged at 470 x g for 5 minutes, and the supernatants were discarded. The cells were collected by resuspending in 200 μL of TRIzol™ reagent (Thermo Fisher Scientific) and stored at -80°C till RNA isolation. RNA was isolated using the RNeasy Mini RNA isolation kit (Qiagen) following the manufacturer’s instructions. The eluted RNA was reverse transcribed to cDNA using Superscript III Reverse transcriptase and random primers (Thermo Fisher Scientific). Quantitative PCR was performed using Power track SYBR green master mix (Applied Biosystems). Forward and reverse primers for the selected genes were designed using the Primer Quest™ tool (IDT). The primers used are listed in **Table 2**. The following amplifying conditions were used: 50°C for 2 minutes, 95°C for 10 minutes, 40 cycles of 95°C for 15 seconds, and 60°C for 1 minute, and a dissociation step of 95°C for 15 seconds, 60°C for 1 minute, 95°C for 15 seconds and 60°C for 15 seconds. The relative gene expression was determined by the 2^-ΔΔCT^ method with RPS9 as the housekeeping gene (63).

**Table 2:** Primer list of select genes near the DAPs for which qPCR was carried out.

S. No	Primer	Gene ID	Primer sequence	Related function
1	*Ifna2*	ENSBTAG 00000046967	Fwd: 5’- GGG AGG TGA GGA AAC AAA GTA A -3’ Rev: 5’- GGA CAA TGG CCT GGG ATA AA -3’	Produced by macrophages and have antiviral activities (54)
2	*Siglec14*	ENSBTAG 00000019227	Fwd: 5’- GAG ACA GAG CCC AGA ATG AAT G -3’ Rev: 5’- CAC GCA GTC ATC TCC ACA AAT A -3’	Glycan-binding protein. Enhances IL-1β release in macrophages (55).
3	*Tnfrsf10d*	ENSBTAG 00000051704	Fwd: 5’- AGT AGA AGA CGG TAG TCA GAG G -3’ Rev: 5’- GGG CTT TCC CAG ACA AGA ATA -3’	Involved in inflammatory response to viral infection (56).
4	*Lrrfip1*	ENSBTAG 00000005354	Fwd: 5’- CTC TAT CCG CCC ACC TAT CTA T -3’ Rev: 5’- GTT GTT CAC TTC GCT GGT TAT G -3’	Anti-viral immunity (57).
5	*Creb3l1*	ENSBTAG 00000006143	Fwd: 5’- CCG TGG AGT CTT CCT TCT TAT C -3’ Rev: 5’- GAG AGA GTC ACG AGC AGT TTA G -3’	Inhibits proliferation of virus-infected cells (58).
6	*Ythdc1*	ENSBTAG 00000015572	Fwd: 5’- GAT GTG TGT GTG TGG CAA ATA G -3’ Rev: 5’- GGT GGC TCA GAT GGT AAA GAA -3’	Regulates viral mRNA splicing (59).
7	*Iqgap2*	ENSBTAG 00000000897	Fwd: 5’- CCT TTC ACC CAC TCC CTA AAC -3’ Rev: 5’- CTG GGT CTT GTT TGT CCT ACT C -3’	Plays a role in interferon response to viruses acting through NFκB pathway (60).
8	*Stk4*	ENSBTAG 00000003257	Fwd: 5’- TTT CAG GGC TTC TCC AAC TAT C -3’ Rev: 5’- CCT CCT CTC ATC CTA CCC ATA A -3’	Regulates immune cell functions in cancer, infection, and autoimmune diseases (61).
9	*Capn2*	ENSBTAG 00000012778	Fwd: 5’- GGC TTC CTT GTC CTT CAC TAT C -3’ Rev: 5’- GGA CAG TGA GCC TTA CCT TAA C -3’	Upregulation is linked to increased aggressiveness of cancer (62).
10	*RPS9*	ENSBTAG 00000006487	Fwd: 5’- GTG AAC ATC CCG TCC TTC AT -3’ Rev: 5’- TCT TGG CGT TCT TCC TCT TC -3’	Small ribosomal subunit protein. Bovine housekeeping gene.

### 2.8 Statistical analysis

GraphPad Prism 9.4.0 was used to plot graphs and perform statistical analysis for cytokine measurements through ELISA and relative gene expression through qPCR. 2-way ANOVA with Sidak’s multiple comparisons test was used for cytokine measurements. An unpaired t-test was used to analyze the qPCR results.

## 3 Results

### 3.1 Altered *in vitro* cytokine responses in bovine γδ T cells following BCG immunization

**Figure 1:**
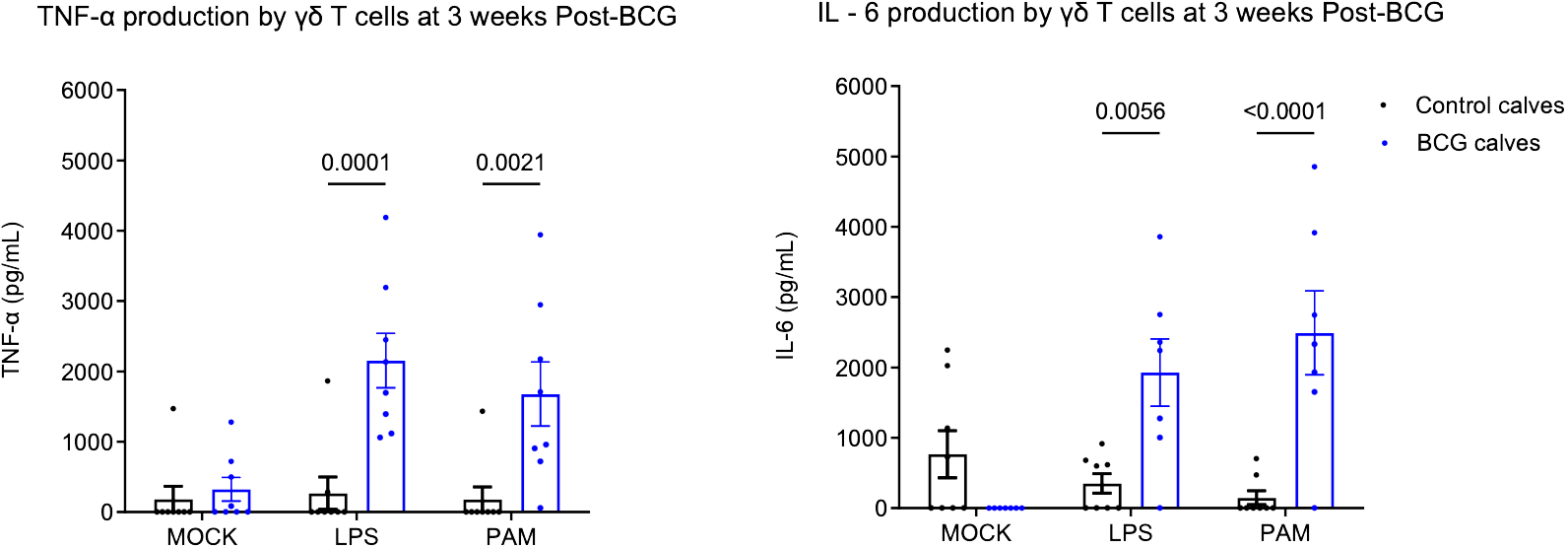
Altered *in vitro* cytokine responses in bovine γδ T cells following BCG immunization. 10^6^ CFU of BCG was administered subcutaneously to preweaned calves in 2 doses at a 2-week interval. The control group remained unvaccinated. Peripheral blood was collected 1 week after the 2^nd^ BCG dose, and γδ T cells were separated by MACS purification. γδ T cells were stimulated *in vitro* with either mock, LPS, or PAM3CSK for 72 hours. Cell culture supernatants were collected, and ELISA was performed to measure innate cytokine production. Data represented as mean ± SEM. P-value as determined by 2-way ANOVA with Sidak’s multiple comparisons test.

Our previous studies showed that BCG administration can induce a trained immune phenotype in calves (7, 8). Bovine γδ T cells comprise a significant subset of immune cells in neonatal calves and play a role in defense against pathogens. To study their ability to undergo trained immunity, we isolated γδ T cells from BCG-immunized and non-immunized preweaned calves and stimulated them *in vitro* with heterologous stimuli such as E. coli LPS, a TLR 4 agonist and PAM3CSK4, a TLR2/1 agonist. We performed ELISA on supernatants to measure proinflammatory cytokine production. On stimulation with *E. coli* LPS or PAM3CSK4, the BCG-treated calves showed enhanced production of TNF α (LPS, P-value = 0.0001 and PAM3CSK4, P-value = 0.0021) and IL-6 cytokines (LPS, P-value = 0.005 and, PAM3CSK4, P-value = <0.0001) (**Figure 1**) 72h after stimulation.

### 3.2 BCG administration differentially reprograms chromatin accessibility in bovine γδ T cells

**Figure 2:**
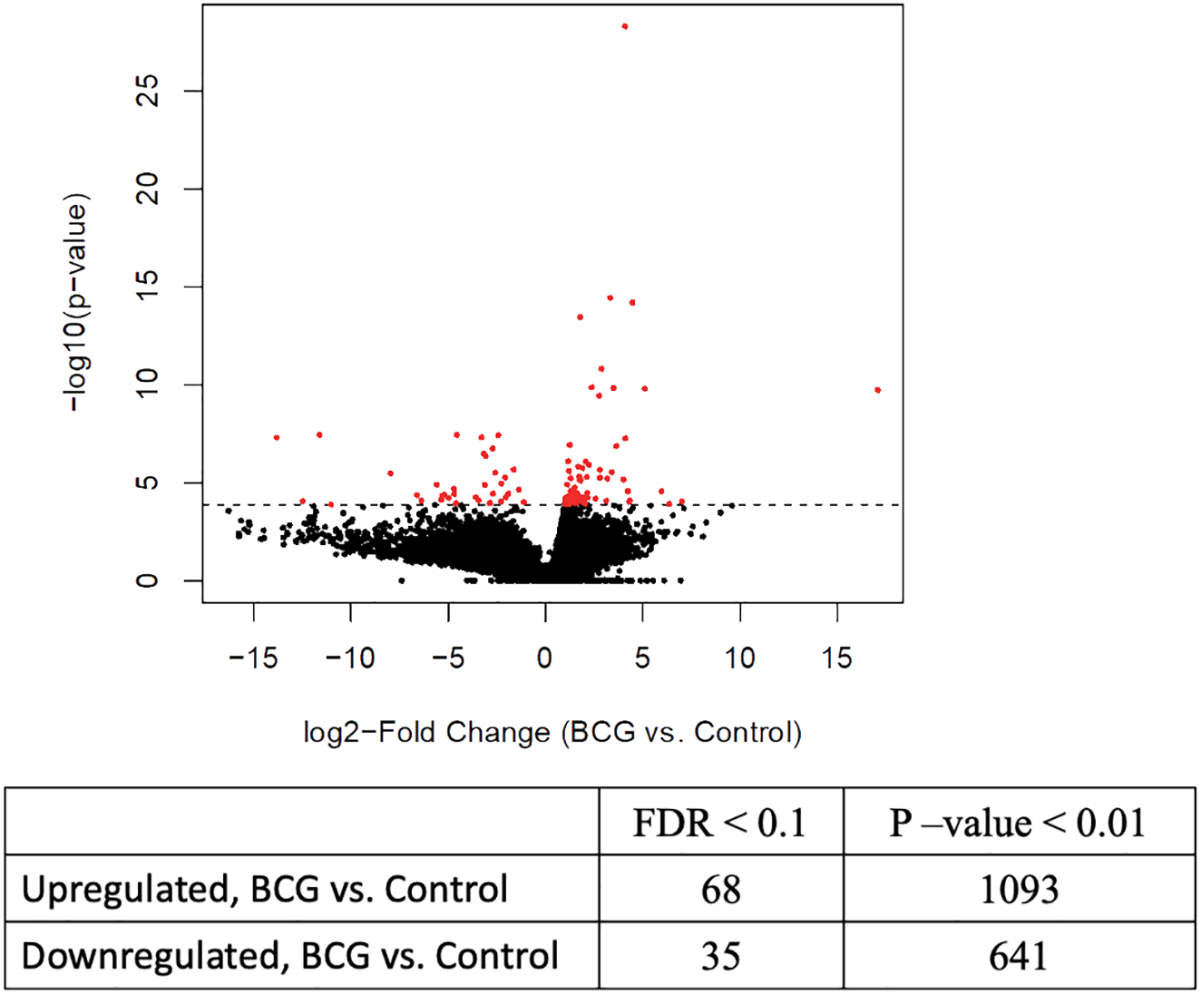
Differential chromatin accessibility in bovine γδ T cells from BCG-immunized calves. ATAC sequencing was performed on γδ T cells from BCG and control calves collected 2 weeks after the 2^nd^ BCG dose, using the Tn5 transposase. Next-gen sequencing was performed on the DNA libraries. After filtering out low-quality reads, peak calling was performed, followed by differential accessible peak analysis of the BCG and control group. The volcano plot shows the results from the DAP analysis with FDR <0.1%.

Previous studies have demonstrated that BCG-induced trained immunity is mediated by genome-wide epigenetic reprogramming; therefore, we were interested in evaluating the epigenetic changes associated with innate training in bovine γδ T cells. We performed ATAC sequencing on γδ T cells from immunized and control calves and observed differential chromatin accessibility in the γδ T cells from BCG-immunized calves. 78,278 consensus peaks were obtained across all samples, and this set was used for differential accessibility analysis. We observed 68 DAPs with increased accessibility and 35 DAPs with decreased accessibility in BCG-immunized versus control calves at a threshold of <0.1 FDR. On a less stringent threshold of P-value <0.01, we observed 1093 increased and 641 decreased DAPs (**Figure 2**). Peak annotation revealed DAPs localized near innate immunity-related genes like *Siglec14*, *Irf4*, *Ifna2*, *Lrrfip1*, and *Tnfrsf10d* with 1 to 4-Log2 fold change increase in γδ T cells from BCG-treated calves compared to cells from unvaccinated calves (**Supplementary Material 4**).

GO enrichment analysis of genes proximal to DAPs can provide insight into downstream pathways that may be impacted by differential accessibility of regulatory elements controlling these genes. The DAPs were annotated to the nearest genes or regulatory elements; distance to the most proximal gene varied from 0 bp to 5 kb. GO enrichment analysis of these nearest genes from DAPs with increased accessibility in BCG-treated samples revealed pathways related to T-cell mediated immune functions such as regulation of IL-2 production and T-cell receptor signaling pathway. The antigen processing and presentation pathway had a high enrichment score (18.2) among the immune function-related pathways. Other pathways that showed high enrichment were the regulation of myeloid leukocyte differentiation, regulation of hemopoiesis, and intracellular signal transduction (**Table 3**).

**Table 3:** Motifs enriched near the open chromatin regions in BCG calves.

Rank	Motif ID	Consensus	p-value	Adjusted p-value	% True positive	% False positive
1	Eomes	NBVRAGGTGTYGSCBN	3.39E-07	3.18E-04	9.52	0.36
2	IRF-5	HACCGAAACYA	2.58E-06	1.01E-03	19.05	3.54
3	ELK-4	BCRCTTCCGGB	1.24E-06	1.91E-03	53.97	25.21
4	ELF-2	AAMCCGGAAGTR	2.45E-06	2.51E-03	36.51	13.01
5	EHF	WACCCGGAAGTA	6.05E-06	3.71E-03	31.75	10.65

Transcription factor binding motif (TFBM) enrichment analysis of differentially accessible peaks can reveal which transcription factors may bind at regulatory elements to influence gene expression. We assessed TFBM enrichment within the sequences within the DAPs. Interestingly, the most enriched TFBMs in peaks with increased accessibility in BCG-treated samples included those for several transcription factors involved in immune gene regulation, such as Eomes and IRF-5 (FDR < 0.1) (**Table 4**).

**Table 4:** GO term enrichment of genes near open regions in BCG calves.

GO enrichment upregulated terms				
S. No	GO term	No of Genes	Enrichment	p-value
1	Antigen processing and presentation of peptide antigen via MHC class Ib	3	18.21	1.65E-04
2	Positive regulation of interleukin-2 production	7	4.72	4.95E-04
3	Regulation of interleukin-2 production	11	4.35	2.91E-05
4	Regulation of myeloid leukocyte differentiation	15	2.68	4.35E-04
5	Antigen processing and presentation of exogenous peptide antigen	18	2.36	2.08E-01
6	T cell receptor signaling pathway	17	2.33	9.76E-04
7	Antigen receptor-mediated signaling pathway	20	2.3	4.12E-04
8	Regulation of leukocyte mediated immunity	19	2.26	7.23E-04
9	Regulation of leukocyte differentiation	28	2.12	1.34E-04
10	Regulation of immune effector process	34	2	8.76E-05
11	Regulation of hemopoiesis	41	1.88	6.90E-05
12	Regulation of immune response	72	1.69	6.98E-06
13	Regulation of immune system process	96	1.44	1.62E-04
14	Intracellular signal transduction	108	1.41	1.17E-04
15	Regulation of response to stimulus	227	1.21	5.42E-04
16	Regulation of cellular metabolic process	319	1.19	4.01E-05
GO enrichment downregulated terms				
S. No	GO term	No of Genes	Enrichment	p-value
1	Langerhans cell differentiation	2	37.97	6.92E-04
2	Inactivation of MAPKK activity	2	37.97	6.92E-04
3	Cell aggregation	5	9.99	1.06E-04
4	Negative regulation of platelet activation	4	8.93	8.58E-04
5	Negative regulation of hemostasis	6	6.9	1.94E-04
6	Negative regulation of response to stimulus	58	1.61	1.71E-04

### 3.3 Gene expression of the genes proximal to DAPs

**Figure 3:**
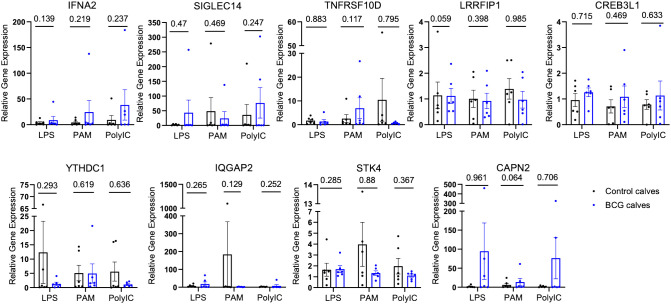
Relative gene expression of select genes proximal to the DAPs. The DAPs were annotated to known genomic features. A few of these genes were selected, and quantitative PCR was performed after *in vitro* stimulation with either mock, LPS, PAM3CSK, or Poly IC/Imiquimod for 4 hours. The CT values were normalized with RPS9, and relative gene expression was determined by the 2^-ΔΔCT^ method. The P-values were obtained by performing an unpaired t-test on the ΔCT values.

To validate the chromatin accessibility results with gene expression, the expression of selected genes near the DAPs was measured by qPCR. Gene expression was compared between BCG-immunized and control calves. The relative gene expression was calculated for each stimulant normalized to the mock (media only). No differences were observed between the control and BCG immunized calves (**Figure 3**).

## 4 Discussion

We have repeatedly observed the capacity for increased proinflammatory cytokine production by PBMCs isolated from cattle with prior exposure to BCG *in vivo* (7, 8). While some of this observation can be explained by trained monocytes (8, 10), our data show that γδ T cells may contribute to this response (**Figure 1**). To our knowledge, ours is the first report of innate training in bovine γδ T cells, although recently, trained immune responses have been reported in human γδ T cells induced by the BCG vaccine (18) and the MMR vaccine (17). Röring et al. (17) recently observed the induction of trained immunity through transcriptomic changes in human γδ T cells; however, no significant difference in innate cytokine production capacity was detected when examining mixed PBMCs. Suen et al. (18) detected distinct functional responses in human γδ T cells following BCG vaccination, with increases in IFN-γ single-producing γδ T cells and TNF/IFN-γ double-producing cells following heterologous stimulation with LPS and *Candida albicans*. Interestingly, however, no differences were observed in the number of circulating γδ T cells or expression of activation markers. In the bovine model, consistent with Suen et al. (18), we observed enhanced TNF-α production by sorted γδ T cells from peripheral blood of BCG-immunized calves in response to stimulation with LPS and PAM3CSK4 (**Figure 1**). We did not evaluate IFN-γ production in the current study, but this will be important to assess in the future.

In cattle, γδ T cells account for a large percentage of the lymphocyte population. They are commonly divided into subsets based on their expression of the surface receptor Workshop Cluster 1 (WC1) (64). WC1 serves as both a pattern recognition receptor and co-receptor on the cell surface. In the current study, we isolated cells from peripheral blood using the monoclonal antibody clone GB21A (mAb clone GB21A), which is specific to the delta chain of the T cell receptor. Therefore, our results include a mixed population of WC1+ and WC1- γδ T cells. Machugh et al. (64) reported that over 50% of the γδ T cells in peripheral blood are WC1+, and a small portion are WC1-. Previous reports have also observed that it is primarily the WC1+ population that responds to BCG vaccination (28, 65, 66); therefore, the enhanced functional response we observed could largely be contributed by the WC1+ cells, although this should be further evaluated in the future.

Innate immune memory is characterized by epigenetic reprogramming, which regulates transcriptional pathways (29). Therefore, we employed ATAC-seq to evaluate changes in chromatin accessibility in γδ T cells from BCG-immunized calves compared to cells from control calves (**Figure 2**). Previous studies have used ATAC-seq to study vaccine-induced epigenetic changes that lead to trained immunity. Investigating the effect of BCG in murine bone marrow-derived macrophages, Kaufmann et al. (37) observed open chromatin regions associated with genes known to be upregulated in response to tuberculosis infection. Brandi et al. (67) observed the enrichment of gene sets related to TNF-α signaling and inflammatory responses near the accessible chromatin regions in human monocytes trained *in vitro* with MV130. Stevens et al. (38) observed genes involved in IFN-α, IFN-γ responses, and IL6/JAK/STAT3 signaling were statistically enriched among genes with altered chromatin accessibility when evaluating epigenetic changes in cDCs from either BCG or DTPw-immunized mice. In γδ T cells from BCG-immunized calves, annotation of the genes proximal to the DAPs in the open chromatin showed up to a 4-log2 FC increase in genes related to immune functions compared to γδ T cells from control calves, including genes related to inflammatory responses and IFN-α responses (**Supplementary Material 4**). Thus, our results are consistent with the previous studies demonstrating a trained immunity signature.

We performed quantitative PCR on several genes proximal to the DAPs that were identified in γδ T cells from BCG-immunized calves, including *Siglec14*, *Tnfrsf10d*, *Ifna2*, *Irf4*, and *Lrrfip1*, to determine if the changes in chromatin accessibility translated to changes in gene expression. However, we observed no significant changes in relative gene expression between the BCG-immunized and control groups in our selected genes (**Figure 3**). One explanation for this disparity could be that although ATAC-seq gives information about chromatin accessibility, it does not indicate whether those genes are expressed or not. Chua et al. (68) highlighted the importance of considering regulatory domains in genome-wide association data, as assigning a DAP to the nearest gene on the physical chromatin is often misleading and lacks prediction power. In a recent report, Moorlag et al. (69) performed a quantitative trait loci (QTL) analysis and observed increased signal of activation mark H3K27ac at *Siglec14* (-5491 bp and -20,552 bp) in human responders to the BCG vaccine compared to non-responders; however, this did not correspond with significant gene expression in human monocytes (69) pointing out that chromatin accessibility does not always guarantee gene transcription.

Despite the lack of gene expression changes, the genes identified by our ATAC-seq data still have implications related to innate training and γδ T cell function. Studying the host genes that influence trained immunity responses in humans by QTL analysis, Moorlag et al. (69) demonstrated the role of *Siglec14* (Sialic acid binding immunoglobulin like lectin - 14) in the positive regulation of trained immunity. This study observed enhanced levels of the activation marks H3K27ac and H3K4me3 at *Siglec14* in monocytes in response to *in vitro* β-glucan treatment, corresponding to the increased inflammatory response in these cells. Evaluation of chromatin marks at *Siglec14* in monocytes from BCG-immunized individuals exposed to attenuated yellow fever virus revealed increased H3K27ac levels in responders to BCG vaccine (individuals with high inflammatory response and low viremia) compared to non-responders (individuals with low inflammatory response and high viremia). In contrast, no such differences were observed in H3K27ac at SIGLEC-5, indicating the importance of SIGLEC-14 in trained immunity responses (69). Although the data from QTL analysis was obtained from whole blood correlating to increased IL-6 production in human monocytes, in bovine γδ T cells, we noted a 4-log2 FC increase (BCG-immunized vs. control calves) in the DAP annotating to the promoter of SIGLEC14 at transcription start site (TSS) of 0 kb (**Supplementary Material 4**). Comparing our result with the response to BCG and β-glucan in human monocytes, we speculate a similar role of *Siglec14* in the enhanced TNF-α and IL-6 cytokine production we observe by the bovine γδ T cells.

Other genes proximal to the DAPs in γδ T cells from BCG-treated calves were *Tnfrsf10d, Ifna2*, and *Irf4* (**Supplementary Material 4**). Shao et al. (56) observed increased *Tnfrsf10d* expression in MERS-CoV-infected human microvascular endothelial cells (HMEC) while studying trained immunity in endothelial cells, suggesting the role of *Tnfrsf10d* receptor in the migration of inflammatory cells to the site of infection. IFNA2 is a key cytokine produced in response to recognizing PAMPs and DAMPs and is known to inhibit viral protein expression in humans (54). Wimmers et al. (57) showed that administration of the H5N1 influenza vaccine to humans induced upregulation of *Irf4* in circulating monocytes. The appearance of genes such as *Siglec14, Tnfrsf10d, Ifna2*, and *Irf4* proximal to the open chromatin regions in the γδ T cells from BCG-immunized calves could suggest their role in influencing epigenetic reprogramming and trained immunity in the bovine model.

Transcription is controlled by transcription factors binding to specific sequence patterns on the DNA known as motifs (35), and observing the motifs enriched in the accessible regions helps to understand key transcription regulators. Motif enrichment analysis of the differentially accessible regions in γδ T cells of BCG-immunized calves showed motifs linked to lymphocyte function and interferon regulatory factors (IRFs). The motif Eomes was highly enriched in the γδ T cells of the BCG-immunized calves (**Table 4**). Eomesodermin (Eomes) is a transcription factor of the T-box family known to play a role in NK cell and CD8 T cell differentiation. Studying the contribution of Eomes in γδ T cells in mice, Lino et al. (70) showed that Eomes+ γδ T cells expressed an effector-like IFNγ-producer CD27+ phenotype pointing to a Th1 lineage.

The motif for IRF-5 was the second most highly enriched motif in the γδ T cells of BCG-immunized calves (**Table 4**). IRF-5 is involved in the expression of proinflammatory cytokines such as type-1 IFNs, IL-6, and TNF-α (71), and it is observed that elevated IRF accessibility drives acute antiviral response in monocytes in infants and young children (72). Similarly, Yamaguchi et al. (73) observed that IFN-stimulated response element-like motifs were more accessible, and transcription factor binding motifs associated with IRFs were enriched in monocytes from individuals receiving consecutive BNT162b2 mRNA vaccinations. Comparing results from these studies with the enriched IRF motif observed in γδ T cells from BCG-treated calves, we speculate that IRF-5 may play a similar role in enhancing innate immune responses. Overall, the motif enrichment analysis shows the contribution of transcription factors responsible for the lymphocyte-related functions and the regulation of proinflammatory cytokines, highlighting the comprehensive nature of γδ T cells.

Gamma delta T cells exhibit innate and adaptive immune characteristics and are thought to serve as a bridge between two arms of the immune system (74, 75). In humans, there are works highlighting the adaptive γδ T cell response to tuberculosis infection, such as an increase in the proportion of γδ T cells in PBMCs (76), expansion of NK-like CD8+ γδ T cells (77), and the role of CDR3 region in recognizing the Mtb antigen (78). Similarly, induction of memory-like γδ T cell response on BCG administration has been observed in humans (79), non-human primates (80), and mice (81). In cattle, vaccination with BCG or infection with virulent *Mycobacterium bovis* induces antigen-specific IFN-γ and IL-17 production by bovine γδ T cells, along with induction of effector and memory cell differentiation in both mucosal and peripheral blood γδ T cells (82). On the other hand, γδ T cells recognize PAMPs through multiple pattern recognition receptors such as TLRs, WC1, and NOD receptors (21, 26), which supports functional innate responses and is consistent with the responses we observe in the current study. We did not restimulate cells with *Mycobacterium bovis* antigen or components of the BCG vaccine in this trial to study the adaptive response. GO enrichment highlighted terms such as ‘intracellular signal transduction,’ ‘regulation of myeloid leukocyte differentiation,’ ‘antigen processing and presentation of peptide antigen via MHC class Ib,’ and ‘antigen processing and presentation of exogenous peptide antigen,’ emphasizing innate response and role in antigen presentation. Conversely, ATAC seq data also revealed adaptive features such as the ‘Eomes’ motif and GO terms like ‘regulation of Interleukin-2 production’ and ‘T cell receptor signaling pathway’ appearing in the analysis (**Tables 3**, **4**). Considering the enhanced innate cytokine production and the ATAC-seq analysis results highlighting both innate and adaptive signatures, we speculate the involvement of both types of responses in calves receiving the BCG vaccine. Further, reports from several recent vaccine trials (73, 83) show that engaging potent trained innate responses while inducing adaptive T-cell responses may be optimum for generating sustained protection against infection.

In conclusion, we observed that bovine γδ T cells adopt a trained immune phenotype following *in vivo* BCG vaccination and changes in chromatin accessibility in regions of genes related to innate immune responses. However, the lack of changes in gene expression that we observed at chosen genes near DAP limits our complete understanding of the mechanism of trained immunity in these cells. Due to material limitations, we could not perform a broader analysis of gene expression such as RNA seq. Our focus for future studies is to involve integrated techniques to obtain more relevant information to connect trained immune responses with epigenetic status, favoring gene transcription and translation. Although results from the current study have limitations, the ATAC-sequencing data obtained can serve as a reference for future works to understand the role of epigenetic status and accessible genes in regulating trained immunity in cattle and nonconventional lymphocyte populations.

## Data availability statement

The data presented in the study are deposited in the NCBI SRA repository, accession numbers SRR29811650 to SRR29811661 with links to BioProject accession number PRJNA1135310.

## Ethics statement

The animal study was approved by Iowa State University Institutional Animal Care and Use Committee. The study was conducted in accordance with the local legislation and institutional requirements.

## Author contributions

BS: Formal analysis, Investigation, Methodology, Writing – original draft, Writing – review & editing. FD: Conceptualization, Investigation, Methodology, Writing – original draft, Writing – review & editing. TM: Conceptualization, Formal analysis, Investigation, Methodology, Writing – original draft, Writing – review & editing. RC: Data curation, Formal analysis, Investigation, Methodology, Software, Visualization, Writing – original draft, Writing – review & editing. CT: Conceptualization, Formal analysis, Supervision, Writing – original draft, Writing – review & editing. JM: Conceptualization, Formal analysis, Project administration, Supervision, Writing – original draft, Writing – review & editing.

## Supplementary material

Supplementary Material 1 Raw data for ATAC-seq DNA library preparation.

Supplementary Material 2 ATAC-seq DNA library traces from TapeStation_batch1.

Supplementary Material 3 ATAC-seq DNA library traces from TapeStation_batch2.

Supplementary Material 4 DAP results BCG vs Control.

Supplementary Material 5 Motif enrichment results.

Supplementary Material 6 GO enrichment results.
