# Supplementary material for: Evidence of innate training in bovine γδ T cells following subcutaneous BCG administration
Source: Front Immunol. 2024 Jul 18;15:1423843. doi: 10.3389/fimmu.2024.1423843 (PMC11295143; doi:10.3389/fimmu.2024.1423843)
Supplement: Supplementary Material 1 — Raw data for ATAC-seq DNA library preparation. [file DataSheet_1.pdf]

Filename: 2022-04-19 - 21.54.37.HSD1000

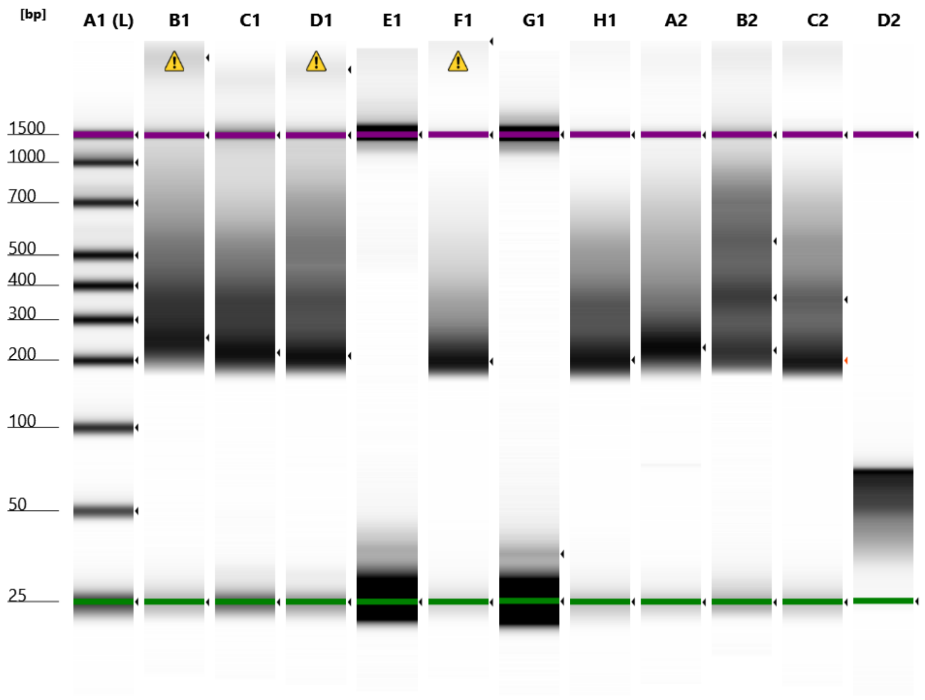

Default image (Contrast 50%), Image is Scaled to Sample

Sample Info

Well	Conc. [pg/µl]	Sample Description	Alert	Observations
A1	2250	Ladder		Ladder
B1	785	238515	⚠	Peak out of Sizing Range
C1	837	238517 -1		
D1	1400	238517 -2	⚠	Peak out of Sizing Range
E1		238522 -1		
F1	5000	238522 -2	⚠	Peak out of Sizing Range
G1	20.6	238523 -1		
H1	3520	238523 -2		
A2	4470	238528		
B2	1960	238534 -1		
C2	5510	238534 -2		
D2		water		

© Agilent Technologies, Inc. 2021

A1: Ladder

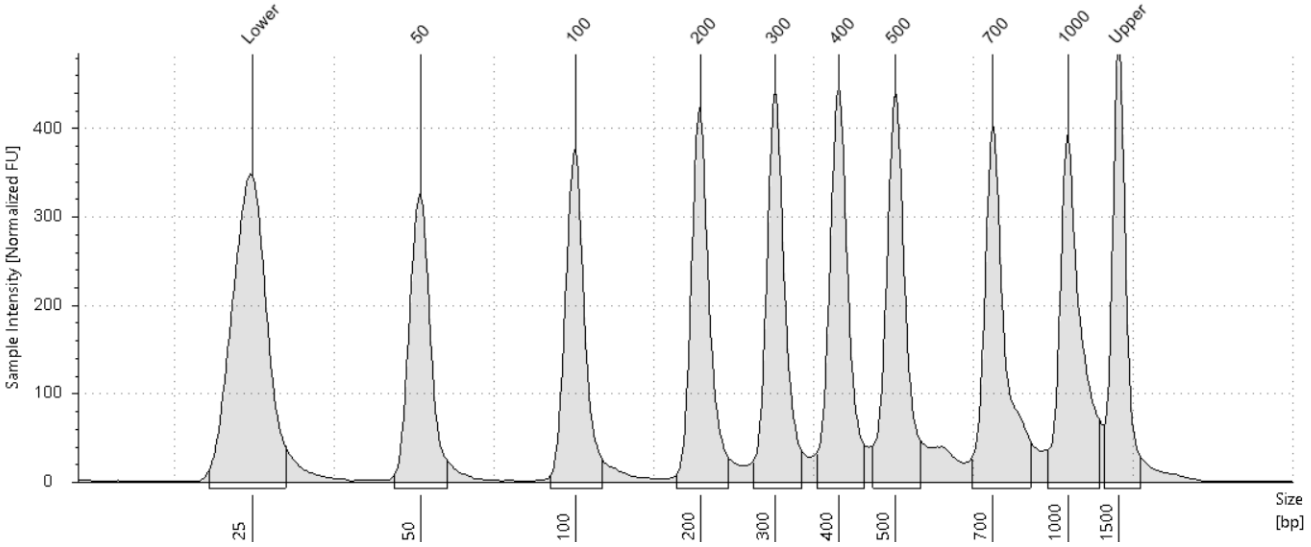

Sample Table

Well	Conc. [pg/µl]	Sample Description	Alert	Observations
A1	2250	Ladder		Ladder

Peak Table

Size [bp]	Calibrated Conc. [pg/µl]	Assigned Conc. [pg/µl]	Peak Molarity [pmol/l]	% Integrated Area	Peak Comment	Observations
25	433	-	26600	-		Lower Marker
50	245	-	7530	10.90		
100	262	-	4030	11.65		
200	275	-	2110	12.22		
300	289	-	1480	12.85		
400	298	-	1150	13.27		
500	296	-	911	13.19		
700	293	-	643	13.03		
1000	289	-	445	12.89		
1500	250	250	256	-		Upper Marker

© Agilent Technologies, Inc. 2021

B1: 238515

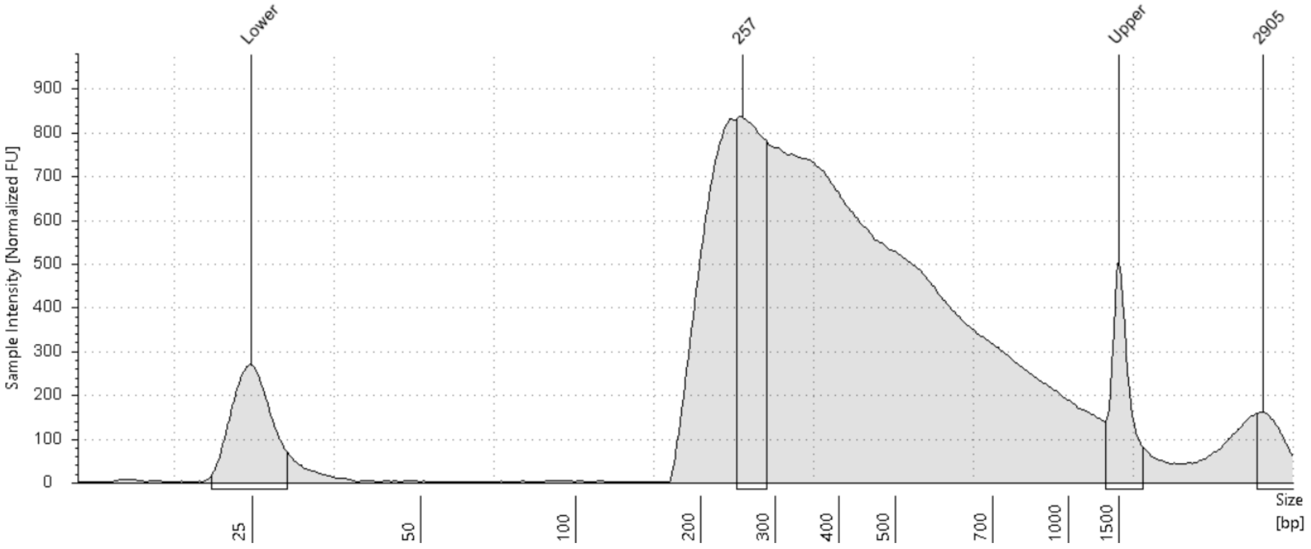

Sample Table

Well	Conc. [pg/µl]	Sample Description	Alert	Observations
B1	785	238515	⚠	Peak out of Sizing Range

Peak Table

Size [bp]	Calibrated Conc. [pg/µl]	Assigned Conc. [pg/µl]	Peak Molarity [pmol/l]	% Integrated Area	Peak Comment	Observations
25	314	-	19300	-		Lower Marker
257	654	-	3920	83.31		
1500	250	250	256	-		Upper Marker
2905	131	-	69.4	16.69		Peak outside of Sizing Range

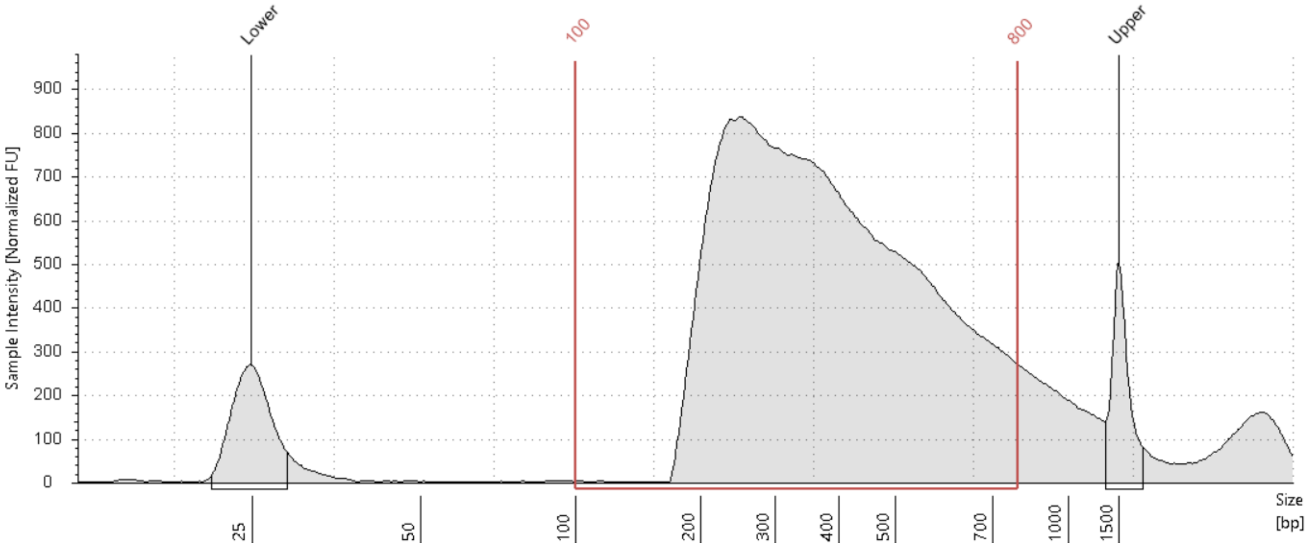

Region Table

From [bp]	To [bp]	Average Size [bp]	Conc. [pg/µl]	Region Molarity [pmol/l]	% of Total	Region Comment	Color
100	800	399	4910	21800	85.48		■

 © Agilent Technologies, Inc. 2021

C1: 238517 -1

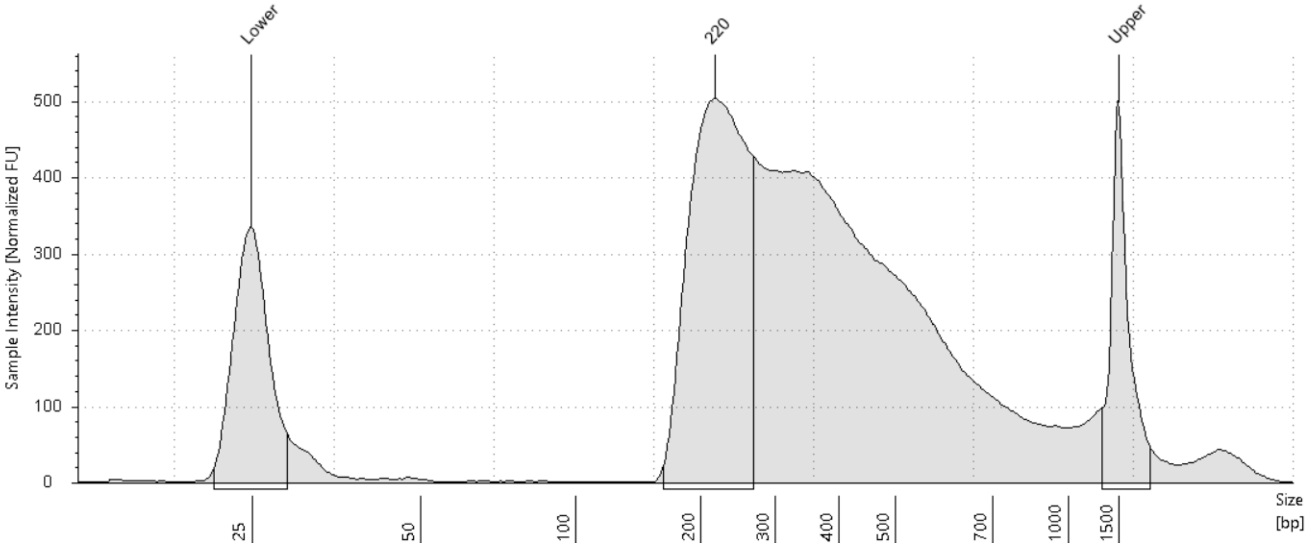

Sample Table

Well	Conc. [pg/µl]	Sample Description	Alert	Observations
C1	837	238517 -1		

Peak Table

Size [bp]	Calibrated Conc. [pg/µl]	Assigned Conc. [pg/µl]	Peak Molarity [pmol/l]	% Integrated Area	Peak Comment	Observations
25	335	-	20600	-		Lower Marker
220	837	-	5870	100.00		
1500	250	250	256	-		Upper Marker

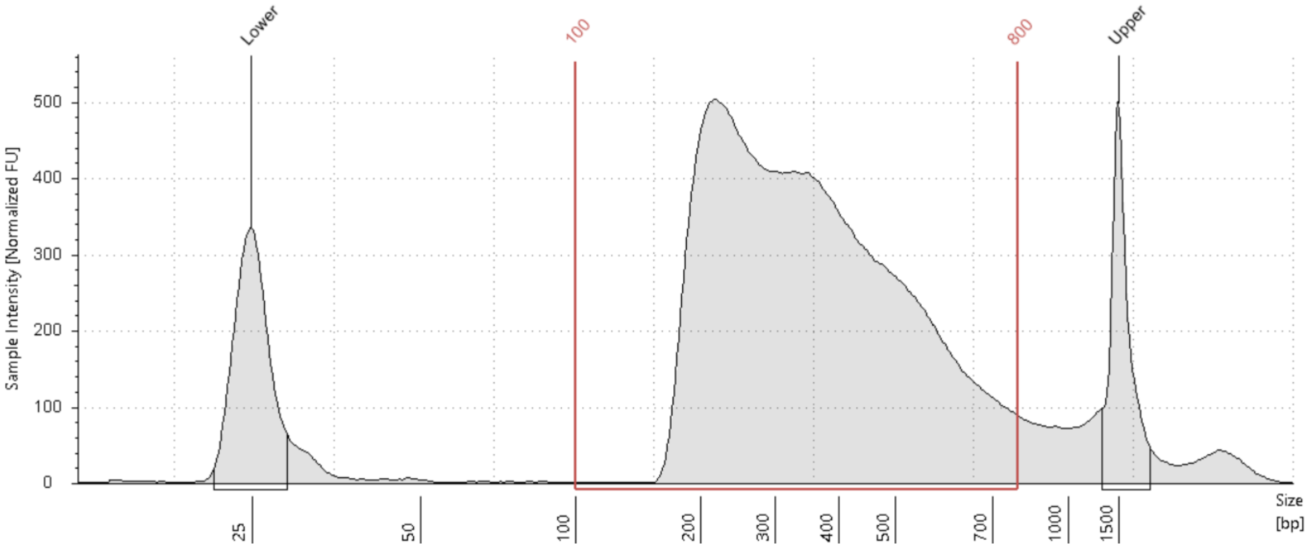

Region Table

From [bp]	To [bp]	Average Size [bp]	Conc. [pg/µl]	Region Molarity [pmol/l]	% of Total	Region Comment	Color
100	800	370	2620	12600	89.80		

© Agilent Technologies, Inc. 2021

D1: 238517 -2

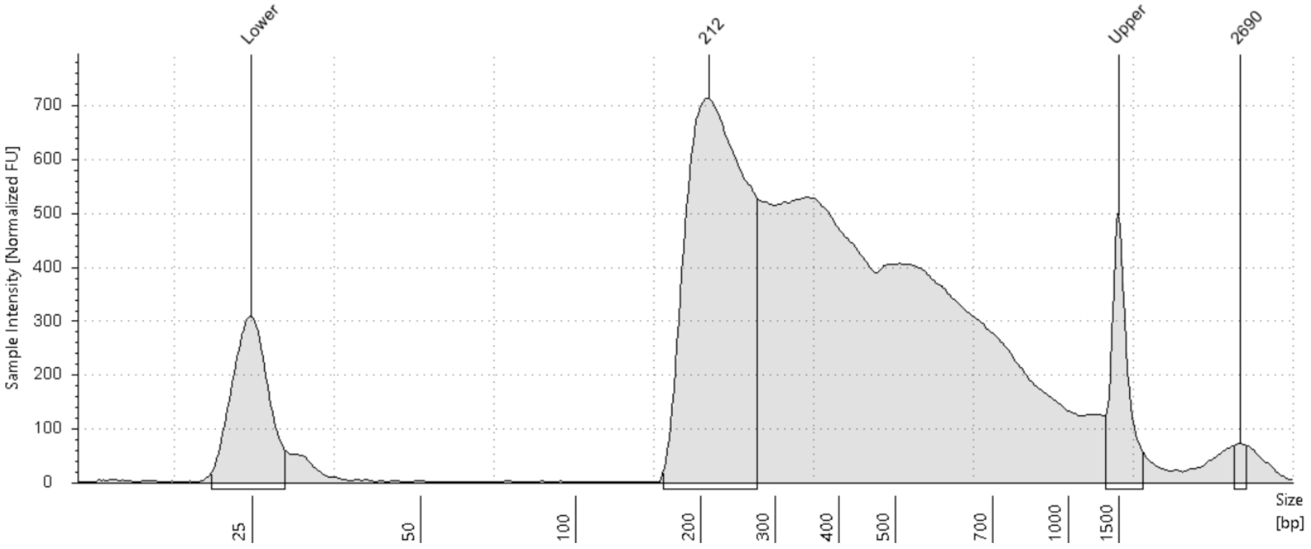

Sample Table

Well	Conc. [pg/µl]	Sample Description	Alert	Observations
D1	1400	238517 -2	⚠	Peak out of Sizing Range

Peak Table

Size [bp]	Calibrated Conc. [pg/µl]	Assigned Conc. [pg/µl]	Peak Molarity [pmol/l]	% Integrated Area	Peak Comment	Observations
25	358	-	22000	-		Lower Marker
212	1380	-	10000	98.15		
1500	250	250	256	-		Upper Marker
2690	26.1	-	14.9	1.85		Peak outside of Sizing Range

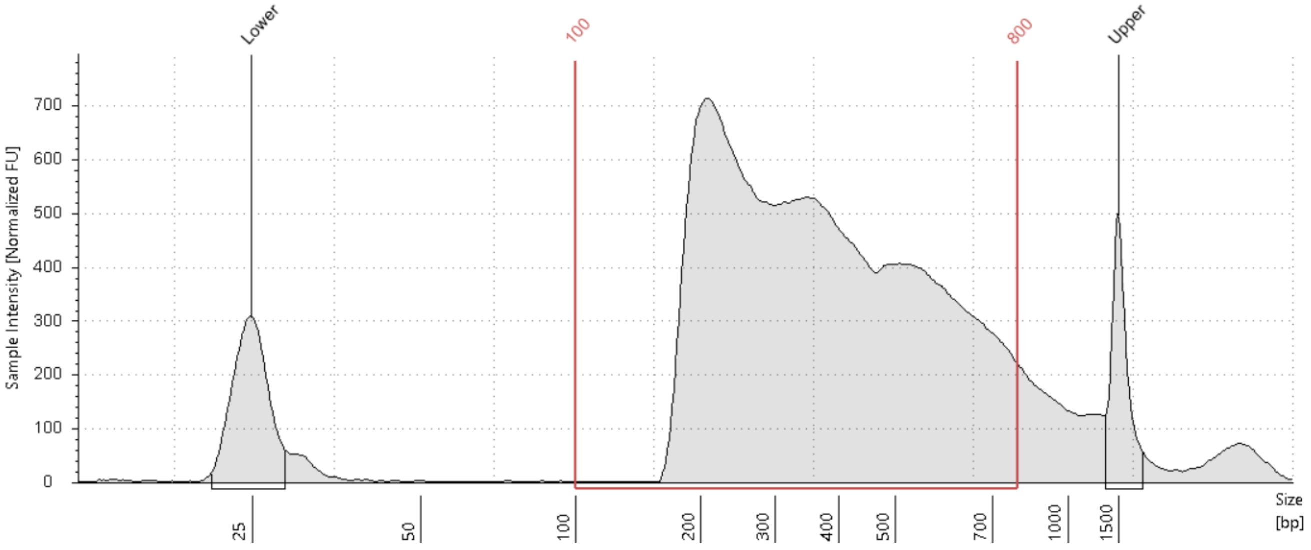

Region Table

From [bp]	To [bp]	Average Size [bp]	Conc. [pg/µl]	Region Molarity [pmol/l]	% of Total	Region Comment	Color
100	800	392	4320	20100	88.33		

E1: 238522 -1

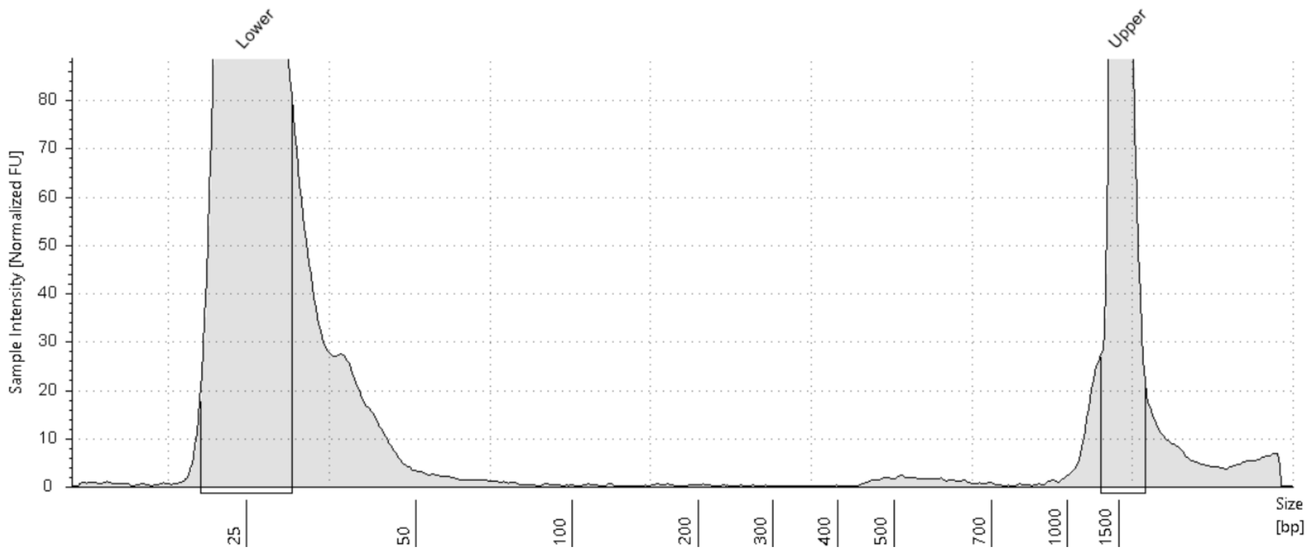

Sample Table

Well	Conc. [pg/µl]	Sample Description	Alert	Observations
E1		238522 -1		

Peak Table

Size [bp]	Calibrated Conc. [pg/µl]	Assigned Conc. [pg/µl]	Peak Molarity [pmol/l]	% Integrated Area	Peak Comment	Observations
25	514	-	31600	-		Lower Marker
1500	250	250	256	-		Upper Marker

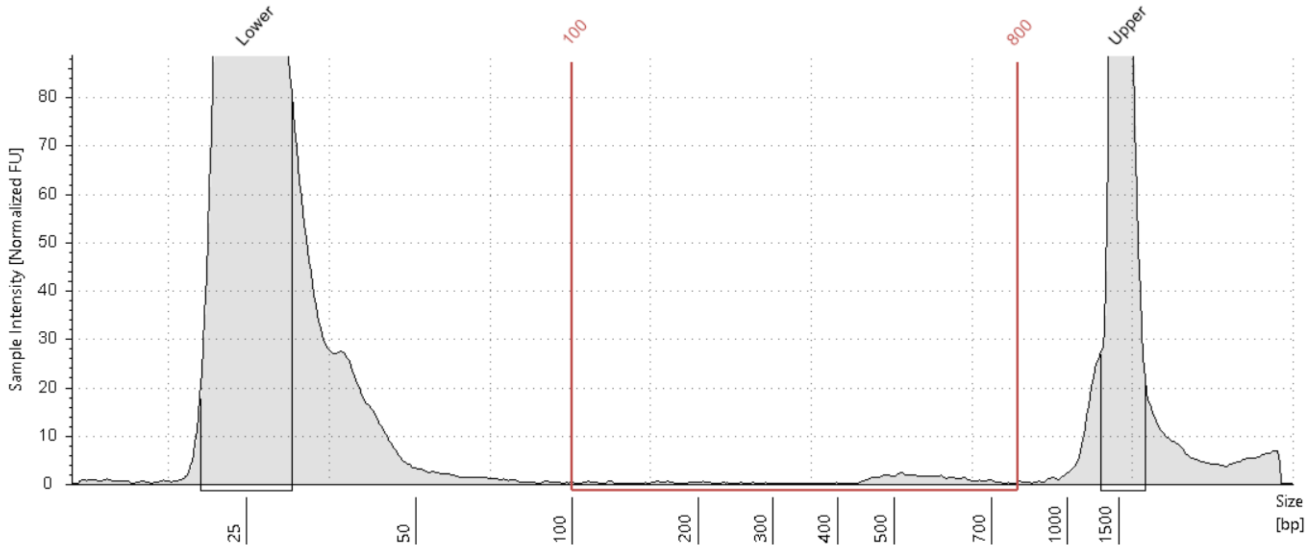

Region Table

From [bp]	To [bp]	Average Size [bp]	Conc. [pg/µl]	Region Molarity [pmol/l]	% of Total	Region Comment	Color
100	800	482	6.05	29.9	4.11		

© Agilent Technologies, Inc. 2021

F1: 238522 -2

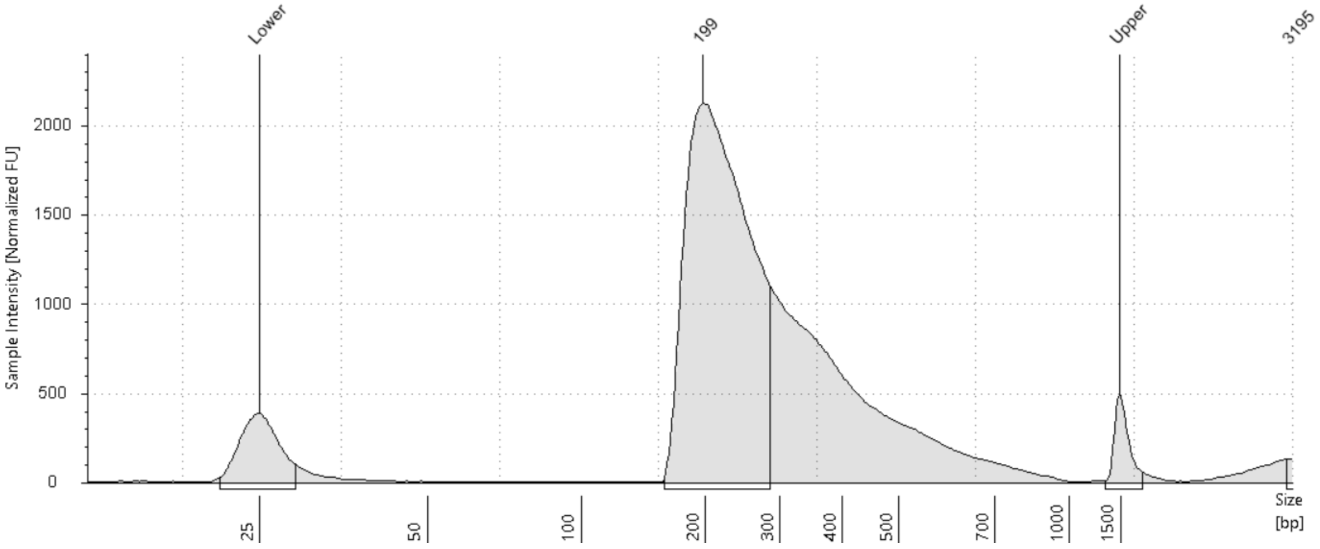

Sample Table

Well	Conc. [pg/µl]	Sample Description	Alert	Observations
F1	5000	238522 -2	⚠	Peak out of Sizing Range

Peak Table

Size [bp]	Calibrated Conc. [pg/µl]	Assigned Conc. [pg/µl]	Peak Molarity [pmol/l]	% Integrated Area	Peak Comment	Observations
25	543	-	33400	-		Lower Marker
199	4900	-	37900	97.92		
1500	250	250	256	-		Upper Marker
3195	104	-	50.0	2.08		Peak outside of Sizing Range

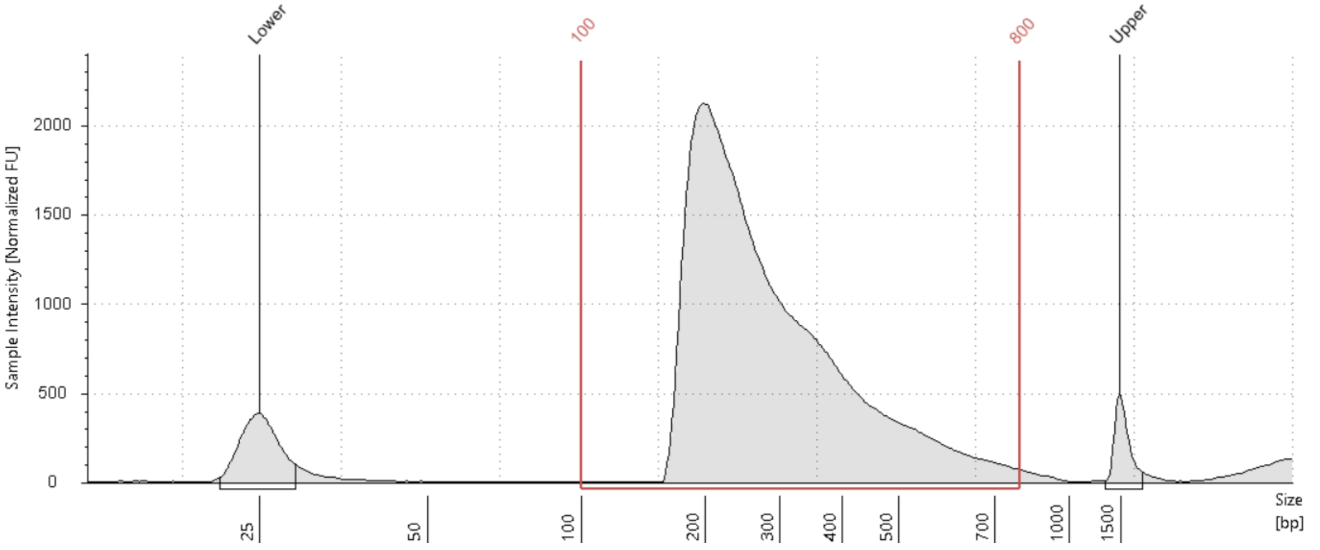

Region Table

From [bp]	To [bp]	Average Size [bp]	Conc. [pg/µl]	Region Molarity [pmol/l]	% of Total	Region Comment	Color
100	800	303	8250	47500	94.61		

© Agilent Technologies, Inc. 2021

G1: 238523 -1

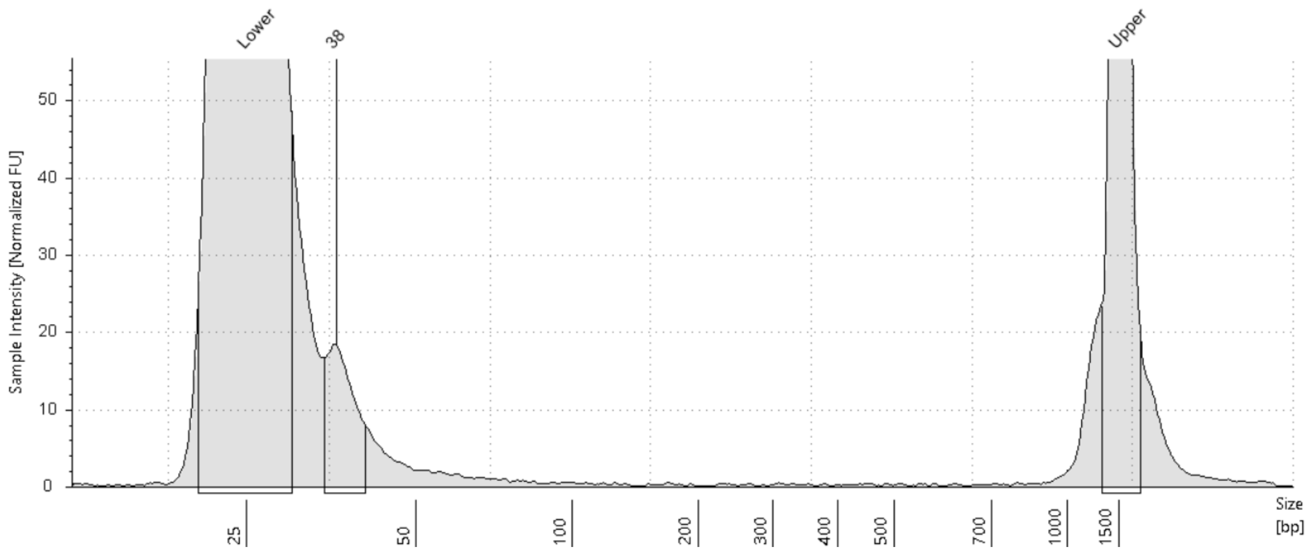

Sample Table

Well	Conc. [pg/µl]	Sample Description	Alert	Observations
Gl	20.6	238523 -1		

Peak Table

Size [bp]	Calibrated Conc. [pg/µl]	Assigned Conc. [pg/µl]	Peak Molarity [pmol/l]	% Integrated Area	Peak Comment	Observations
25	694	-	42700	-		Lower Marker
38	20.6	-	831	100.00		
1500	250	250	256	-		Upper Marker

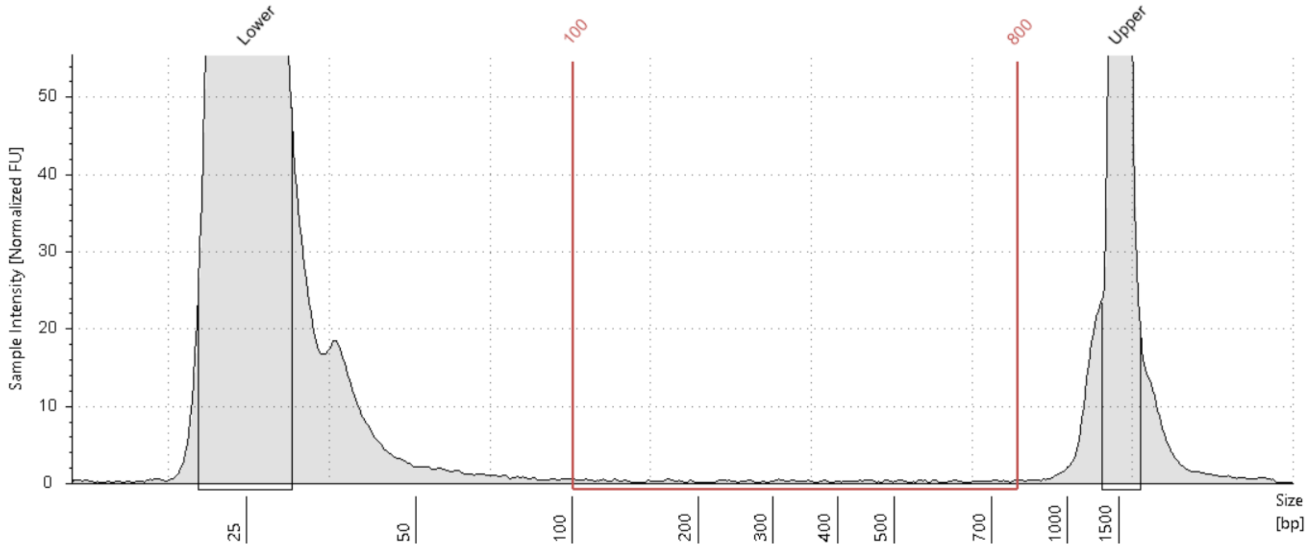

Region Table

From [bp]	To [bp]	Average Size [bp]	Conc. [pg/µl]	Region Molarity [pmol/l]	% of Total	Region Comment	Color
100	800	357	2.80	23.6	2.82		

© Agilent Technologies, Inc. 2021

H1: 238523 -2

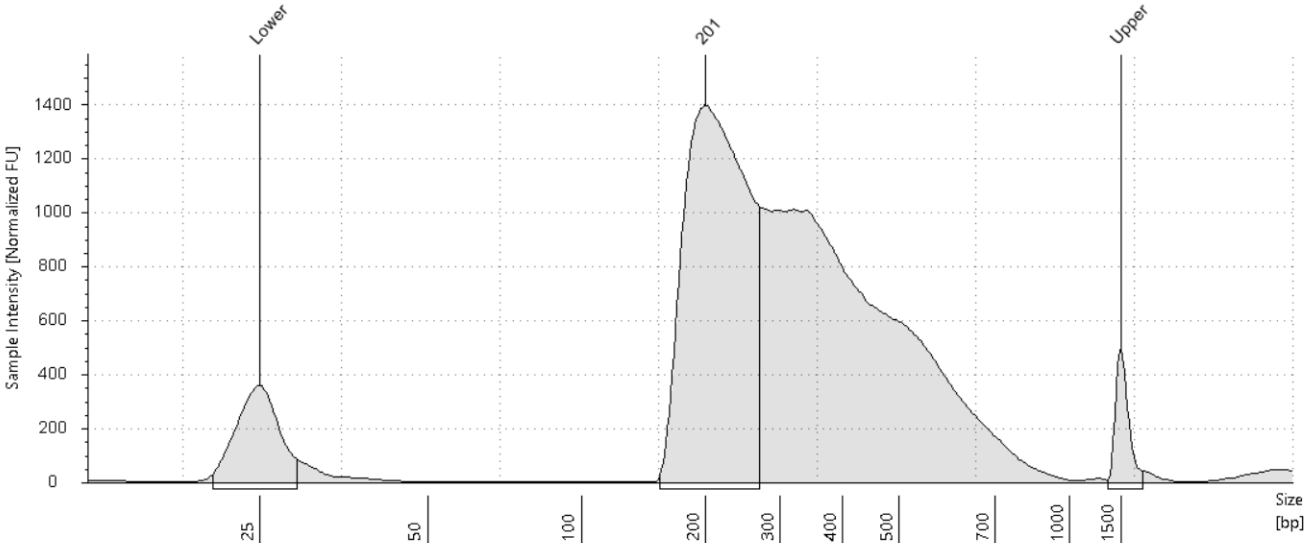

Sample Table

Well	Conc. [pg/µl]	Sample Description	Alert	Observations
H1	3520	238523 -2		

Peak Table

Size [bp]	Calibrated Conc. [pg/µl]	Assigned Conc. [pg/µl]	Peak Molarity [pmol/l]	% Integrated Area	Peak Comment	Observations
25	603	-	37100	-		Lower Marker
201	3520	-	27000	100.00		
1500	250	250	256	-		Upper Marker

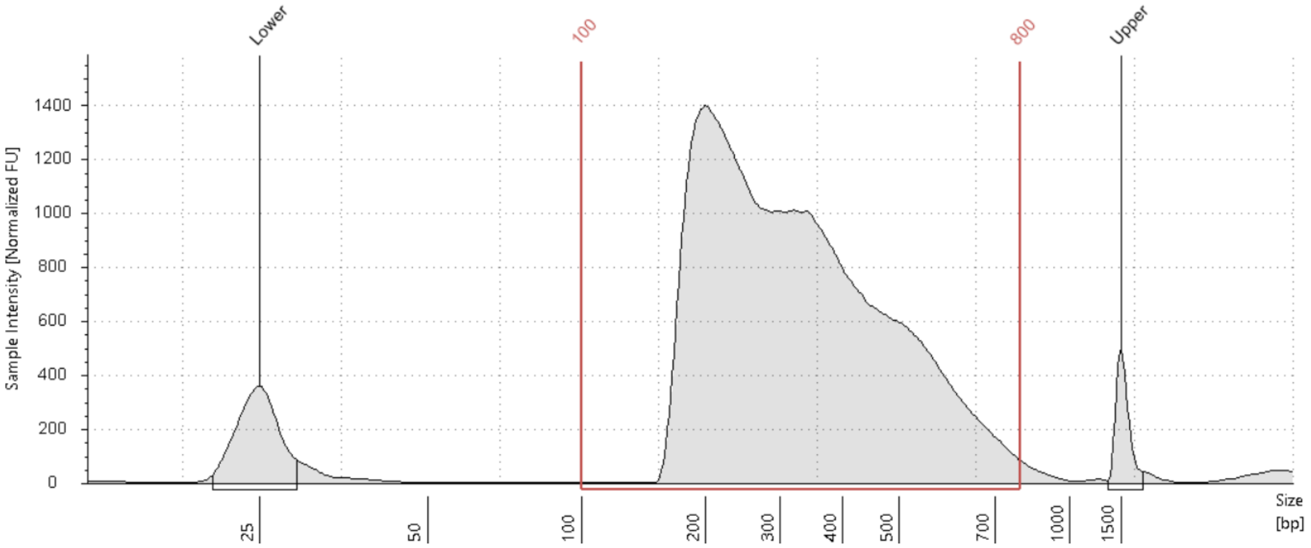

Region Table

From [bp]	To [bp]	Average Size [bp]	Conc. [pg/µl]	Region Molarity [pmol/l]	% of Total	Region Comment	Color
100	800	344	8890	46000	96.64		

© Agilent Technologies, Inc. 2021

A2: 238528

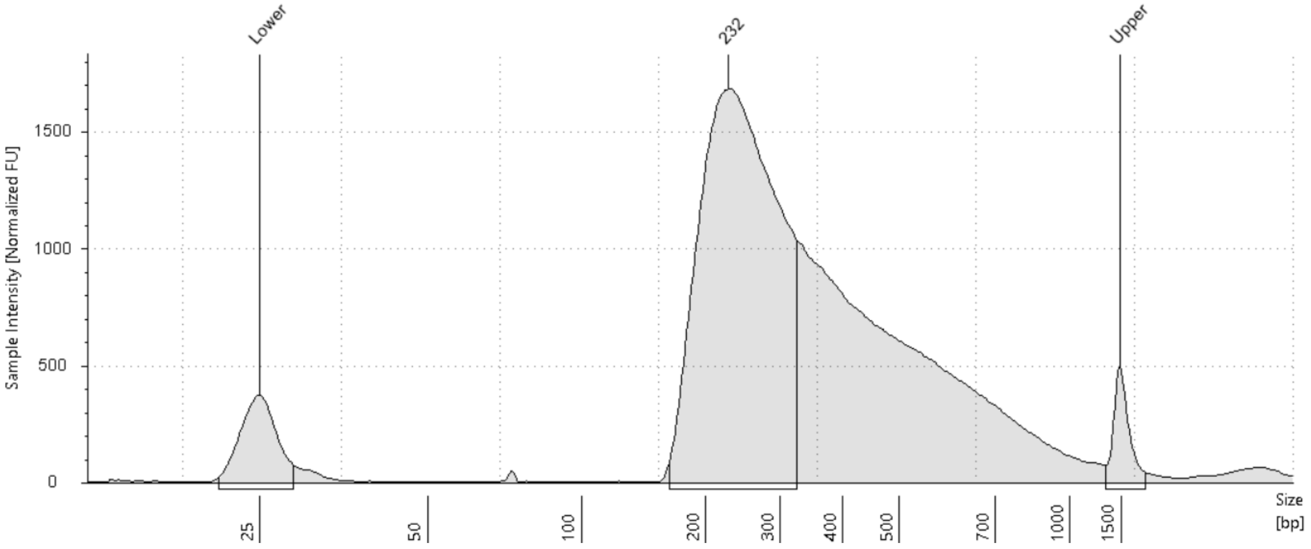

Sample Table

Well	Conc. [pg/µl]	Sample Description	Alert	Observations
A2	4470	238528		

Peak Table

Size [bp]	Calibrated Conc. [pg/µl]	Assigned Conc. [pg/µl]	Peak Molarity [pmol/l]	% Integrated Area	Peak Comment	Observations
25	450	-	27700	-		Lower Marker
232	4470	-	29600	100.00		
1500	250	250	256	-		Upper Marker

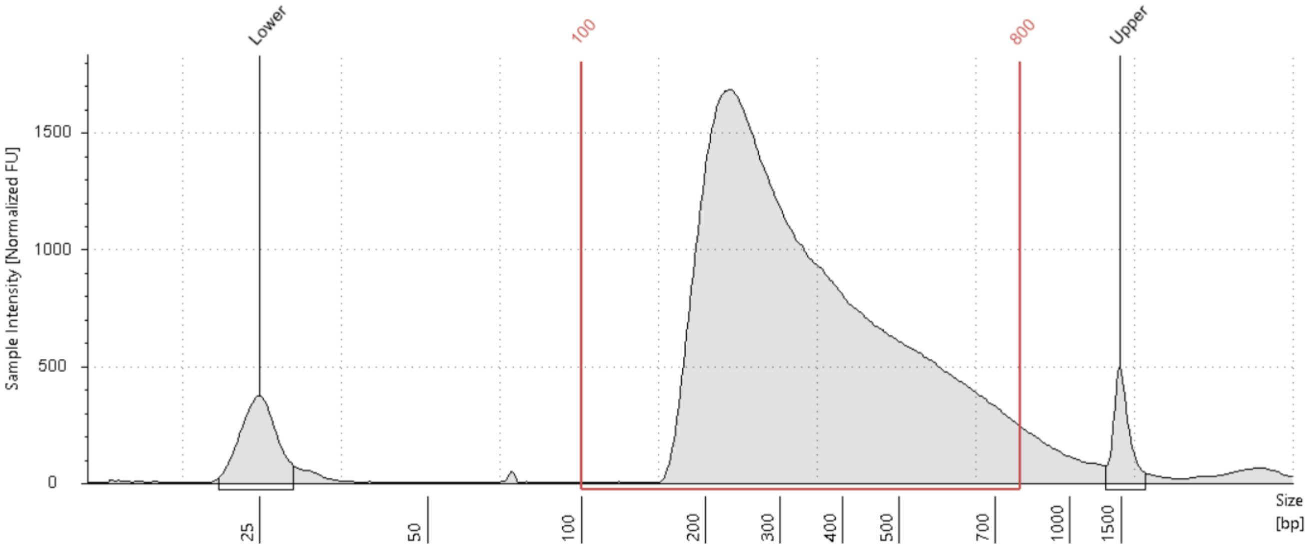

Region Table

From [bp]	To [bp]	Average Size [bp]	Conc. [pg/µl]	Region Molarity [pmol/l]	% of Total	Region Comment	Color
100	800	359	8330	41500	93.75		

© Agilent Technologies, Inc. 2021

B2: 238534 -1

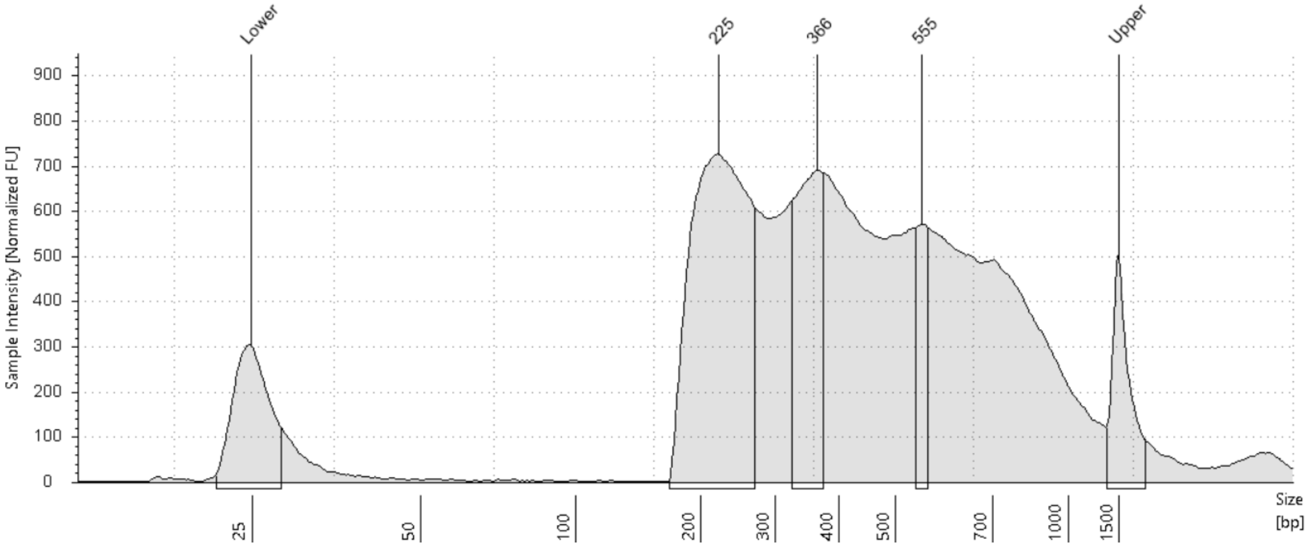

Sample Table

Well	Conc. [pg/µl]	Sample Description	Alert	Observations
B2	1960	238534 -1		

Peak Table

Size [bp]	Calibrated Conc. [pg/µl]	Assigned Conc. [pg/µl]	Peak Molarity [pmol/l]	% Integrated Area	Peak Comment	Observations
25	320	-	19700	-		Lower Marker
225	1240	-	8510	63.57		
366	529	-	2230	27.02		
555	184	-	511	9.41		
1500	250	250	256	-		Upper Marker

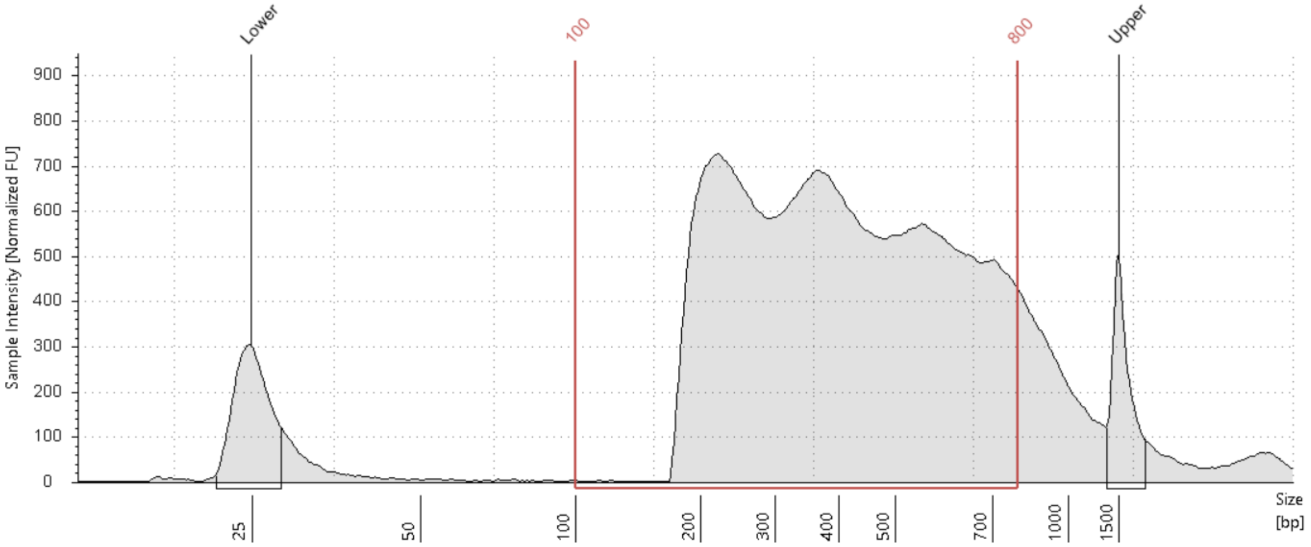

Region Table

From [bp]	To [bp]	Average Size [bp]	Conc. [pg/µl]	Region Molarity [pmol/l]	% of Total	Region Comment	Color
100	800	421	5010	21700	85.61		

 © Agilent Technologies, Inc. 2021

C2: 238534 -2

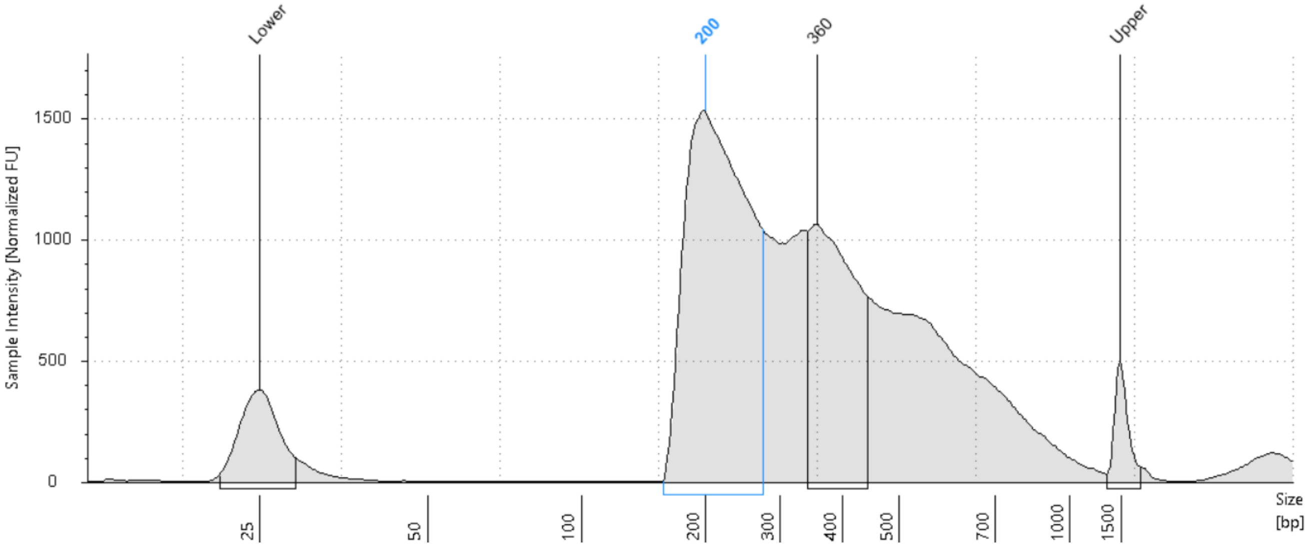

Sample Table

Well	Conc. [pg/µl]	Sample Description	Alert	Observations
C2	5510	238534 -2		

Peak Table

Size [bp]	Calibrated Conc. [pg/µl]	Assigned Conc. [pg/µl]	Peak Molarity [pmol/l]	% Integrated Area	Peak Comment	Observations
25	564	-	34700	-		Lower Marker
200	3640	-	27900	66.01		
360	1870	-	8000	33.99		
1500	250	250	256	-		Upper Marker

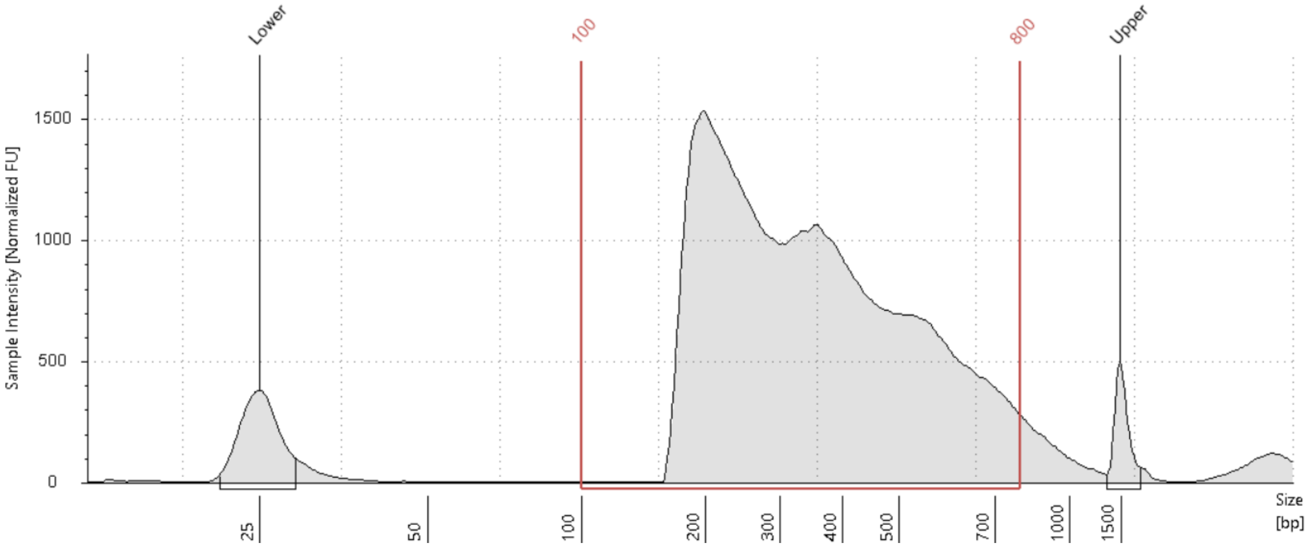

Region Table

From [bp]	To [bp]	Average Size [bp]	Conc. [pg/µl]	Region Molarity [pmol/l]	% of Total	Region Comment	Color
100	800	368	9650	47700	93.10		

© Agilent Technologies, Inc. 2021

D2: water

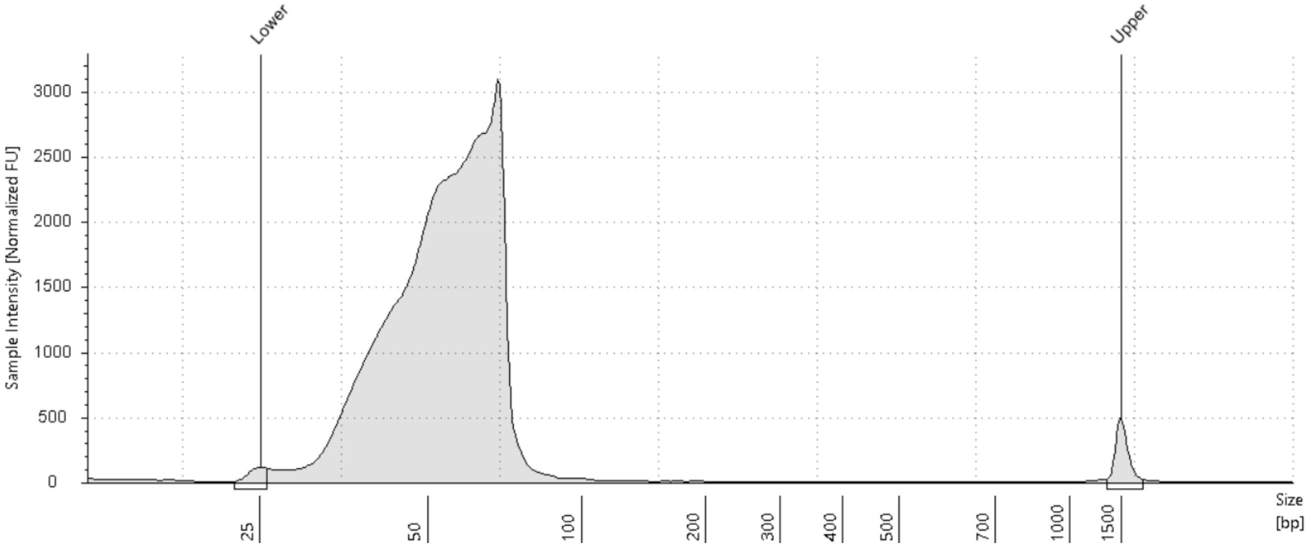

Sample Table

Well	Conc. [pg/µl]	Sample Description	Alert	Observations
D2		water		

Peak Table

Size [bp]	Calibrated Conc. [pg/µl]	Assigned Conc. [pg/µl]	Peak Molarity [pmol/l]	% Integrated Area	Peak Comment	Observations
25	82.2	-	5060	-		Lower Marker
1500	250	250	256	-		Upper Marker

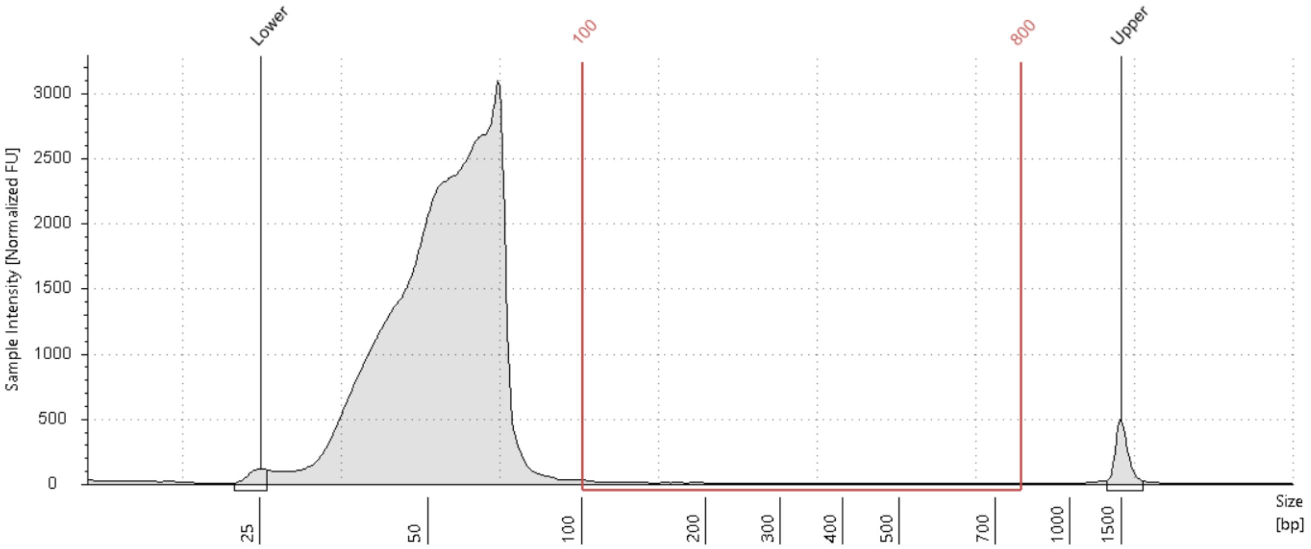

Region Table

From [bp]	To [bp]	Average Size [bp]	Conc. [pg/µl]	Region Molarity [pmol/l]	% of Total	Region Comment	Color
100	800	255	81.0	808	0.70		

© Agilent Technologies, Inc. 2021
