# Supplementary material for: Evidence of innate training in bovine γδ T cells following subcutaneous BCG administration
Source: Front Immunol. 2024 Jul 18;15:1423843. doi: 10.3389/fimmu.2024.1423843 (PMC11295143; doi:10.3389/fimmu.2024.1423843)
Supplement: Supplementary Material 2 — ATAC-seq DNA library traces from TapeStation_batch1. [file DataSheet_2.pdf]

Filename: 2022-06-03 - 21.53.27.HSD1000

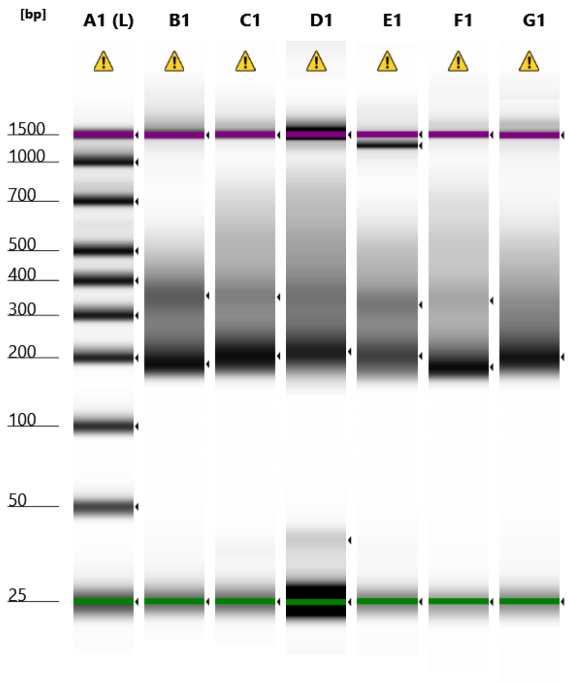

Default image (Contrast 50%), Image is Scaled to Sample

Sample Info

Well	Conc. [pg/µl]	Sample Description	Alert	Observations
A1	2400	Ladder	⚠	Caution! Expired ScreenTape device; Ladder
B1	1250	238530	⚠	Caution! Expired ScreenTape device
C1	1060	238535	⚠	Caution! Expired ScreenTape device
D1	134	238538	⚠	Caution! Expired ScreenTape device
E1	3790	238543	⚠	Caution! Expired ScreenTape device
F1	1490	238544	⚠	Caution! Expired ScreenTape device
G1	908	238545	⚠	Caution! Expired ScreenTape device

© Agilent Technologies, Inc. 2021

A1: Ladder

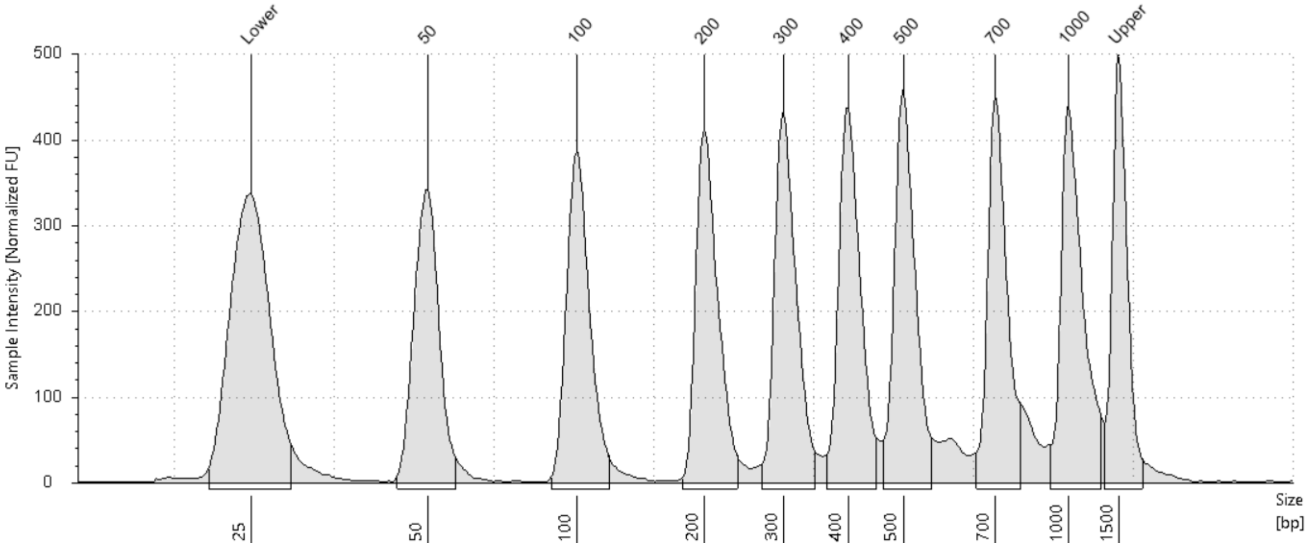

Sample Table

Well	Conc. [pg/µl]	Sample Description	Alert	Observations
A1	2400	Ladder	⚠	Caution! Expired ScreenTape device; Ladder

Peak Table

Size [bp]	Calibrated Conc. [pg/µl]	Assigned Conc. [pg/µl]	Peak Molarity [pmol/l]	% Integrated Area	Peak Comment	Observations
25	444	-	27300	-		Lower Marker
50	281	-	8650	11.70		
100	298	-	4580	12.40		
200	297	-	2280	12.37		
300	307	-	1570	12.78		
400	310	-	1190	12.93		
500	312	-	959	12.98		
700	282	-	619	11.73		
1000	315	-	484	13.11		
1500	250	250	256	-		Upper Marker

© Agilent Technologies, Inc. 2021

B1: 238530

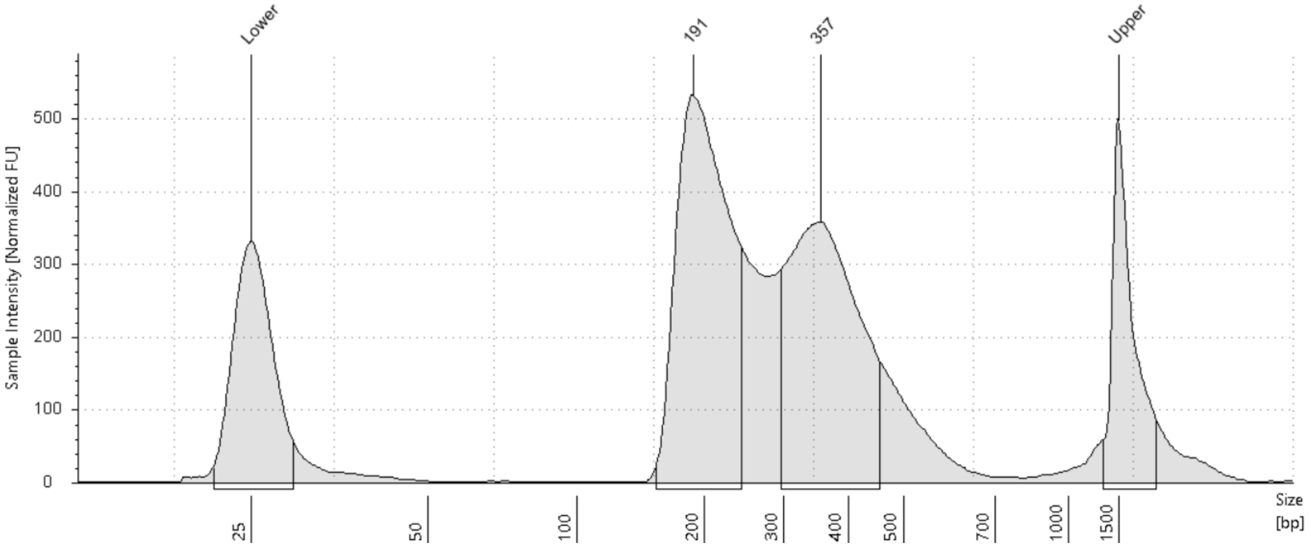

Sample Table

Well	Conc. [pg/µl]	Sample Description	Alert	Observations
B1	1250	238530	⚠	Caution! Expired ScreenTape device

Peak Table

Size [bp]	Calibrated Conc. [pg/µl]	Assigned Conc. [pg/µl]	Peak Molarity [pmol/l]	% Integrated Area	Peak Comment	Observations
25	313	-	19300	-		Lower Marker
191	653	-	5260	52.06		
357	602	-	2590	47.94		
1500	250	250	256	-		Upper Marker

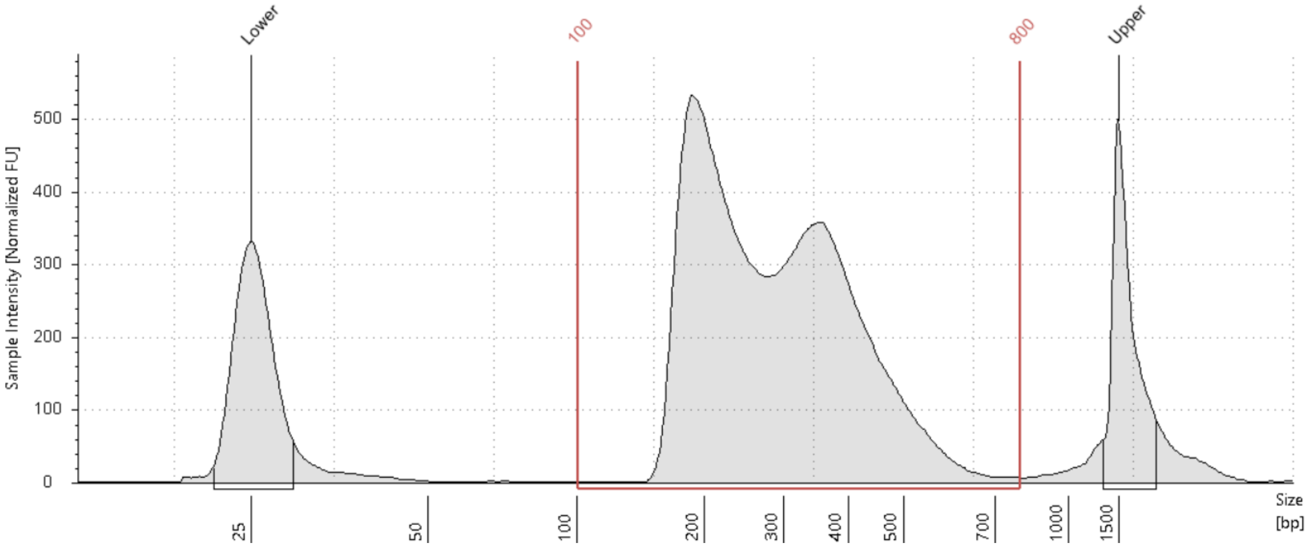

Region Table

From [bp]	To [bp]	Average Size [bp]	Conc. [pg/µl]	Region Molarity [pmol/l]	% of Total	Region Comment	Color
100	800	303	1640	9370	92.70		

© Agilent Technologies, Inc. 2021

C1: 238535

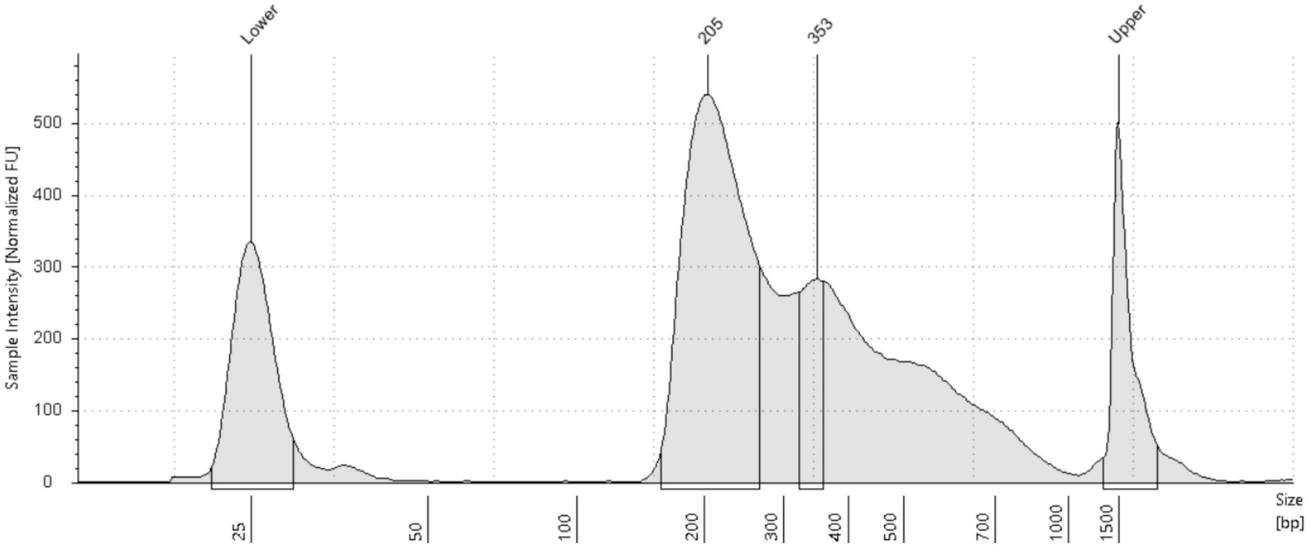

Sample Table

Well	Conc. [pg/µl]	Sample Description	Alert	Observations
C1	1060	238535	⚠	Caution! Expired ScreenTape device

Peak Table

Size [bp]	Calibrated Conc. [pg/µl]	Assigned Conc. [pg/µl]	Peak Molarity [pmol/l]	% Integrated Area	Peak Comment	Observations
25	373	-	22900	-		Lower Marker
205	898	-	6750	84.64		
353	163	-	710	15.36		
1500	250	250	256	-		Upper Marker

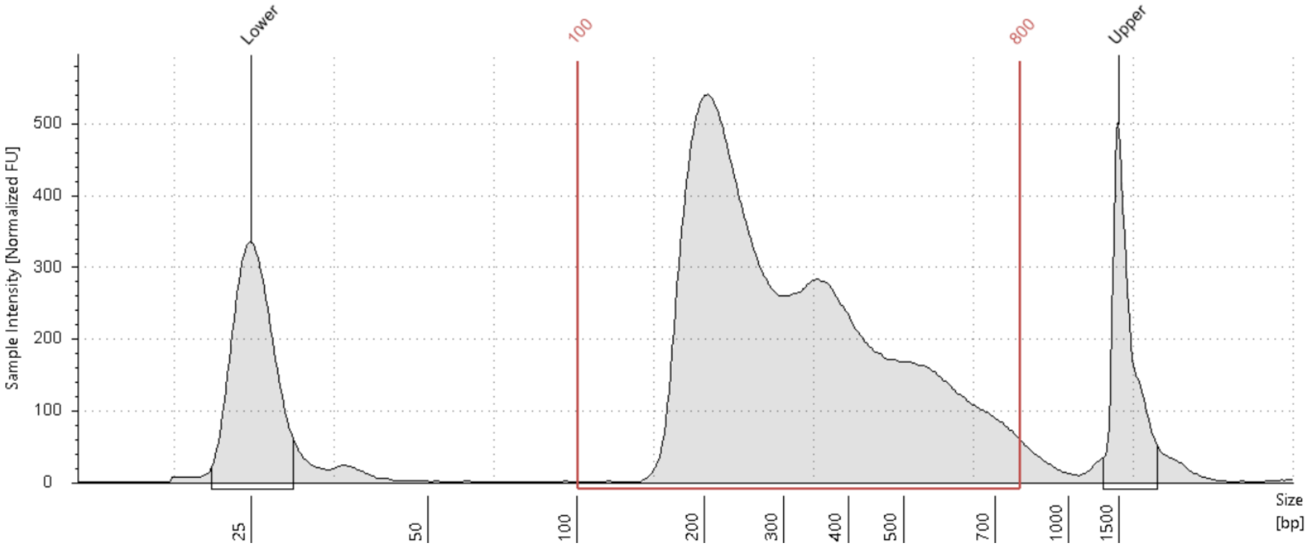

Region Table

From [bp]	To [bp]	Average Size [bp]	Conc. [pg/µl]	Region Molarity [pmol/l]	% of Total	Region Comment	Color
100	800	339	2020	10800	93.64		

© Agilent Technologies, Inc. 2021

D1: 238538

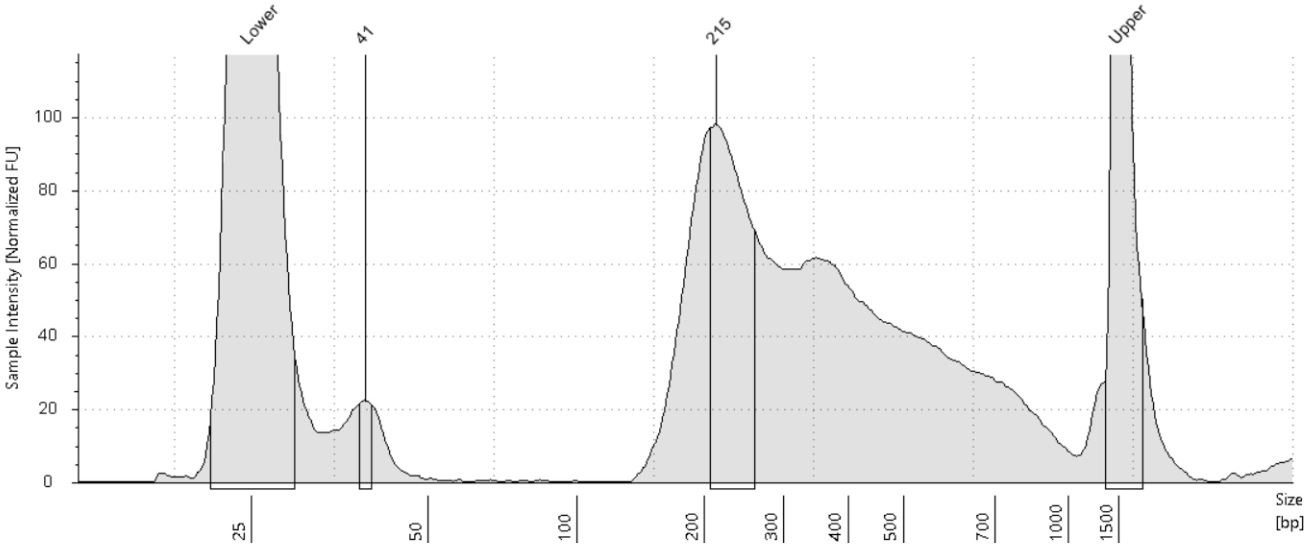

Sample Table

Well	Conc. [pg/µl]	Sample Description	Alert	Observations
D1	134	238538	⚠	Caution! Expired ScreenTape device

Peak Table

Size [bp]	Calibrated Conc. [pg/µl]	Assigned Conc. [pg/µl]	Peak Molarity [pmol/l]	% Integrated Area	Peak Comment	Observations
25	425	-	26100	-		Lower Marker
41	8.86	-	331	6.63		
215	125	-	893	93.37		
1500	250	250	256	-		Upper Marker

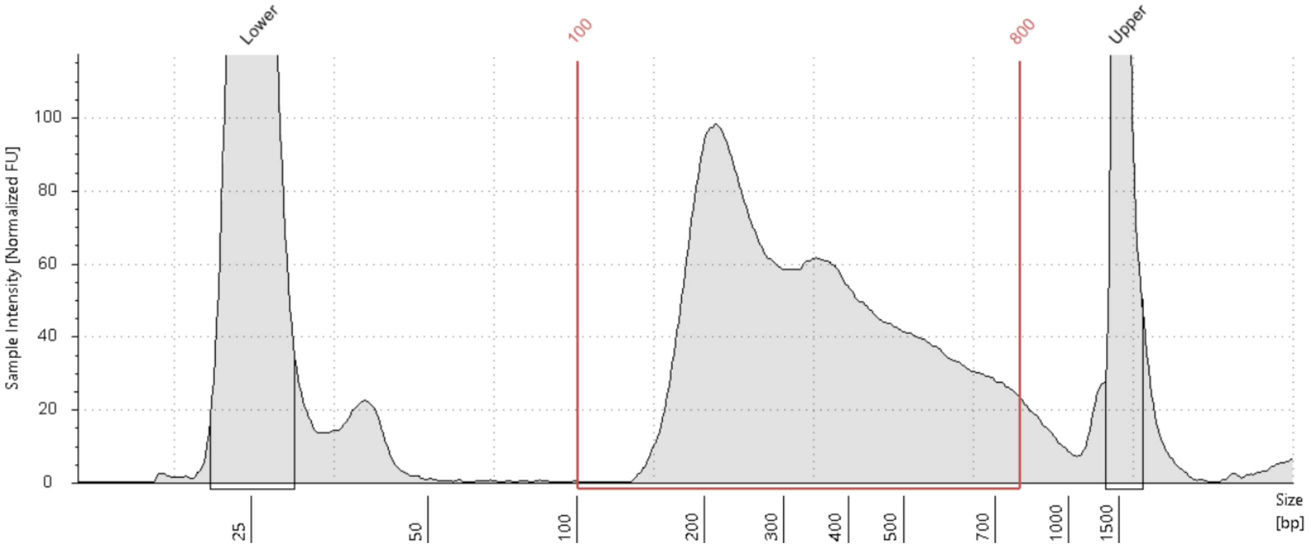

Region Table

From [bp]	To [bp]	Average Size [bp]	Conc. [pg/µl]	Region Molarity [pmol/l]	% of Total	Region Comment	Color
100	800	362	602	3060	82.07		

© Agilent Technologies, Inc. 2021

E1: 238543

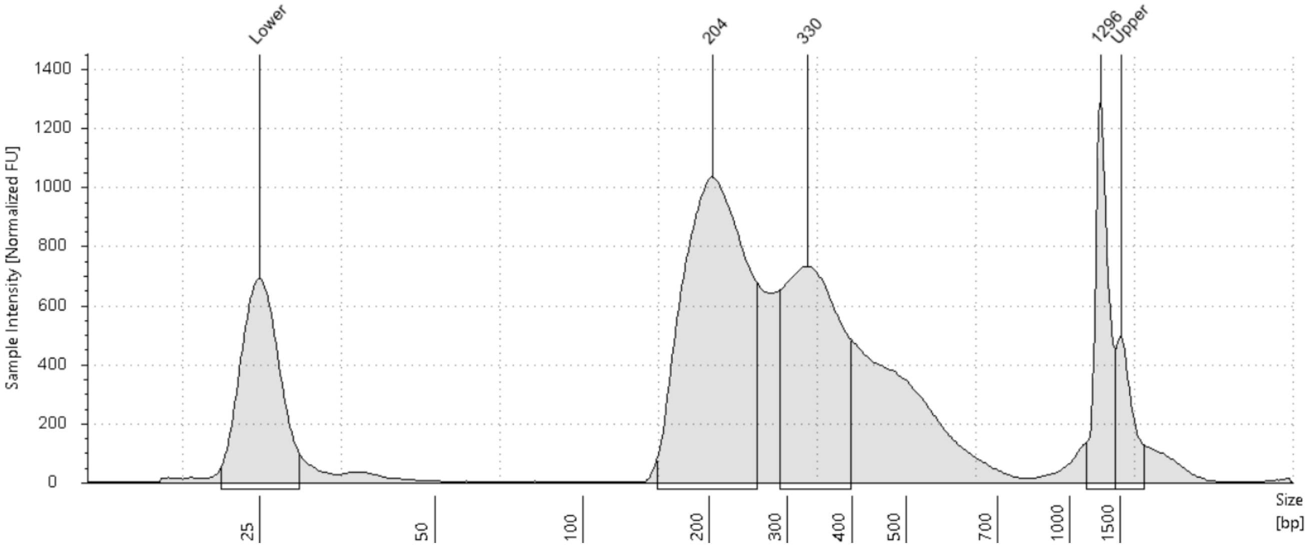

Sample Table

Well	Conc. [pg/µl]	Sample Description	Alert	Observations
E1	3790	238543	⚠	Caution! Expired ScreenTape device

Peak Table

Size [bp]	Calibrated Conc. [pg/µl]	Assigned Conc. [pg/µl]	Peak Molarity [pmol/l]	% Integrated Area	Peak Comment	Observations
25	822	-	50600	-		Lower Marker
204	1990	-	15000	52.59		
330	1260	-	5890	33.33		
1296	533	-	633	14.08		
1500	250	250	256	-		Upper Marker

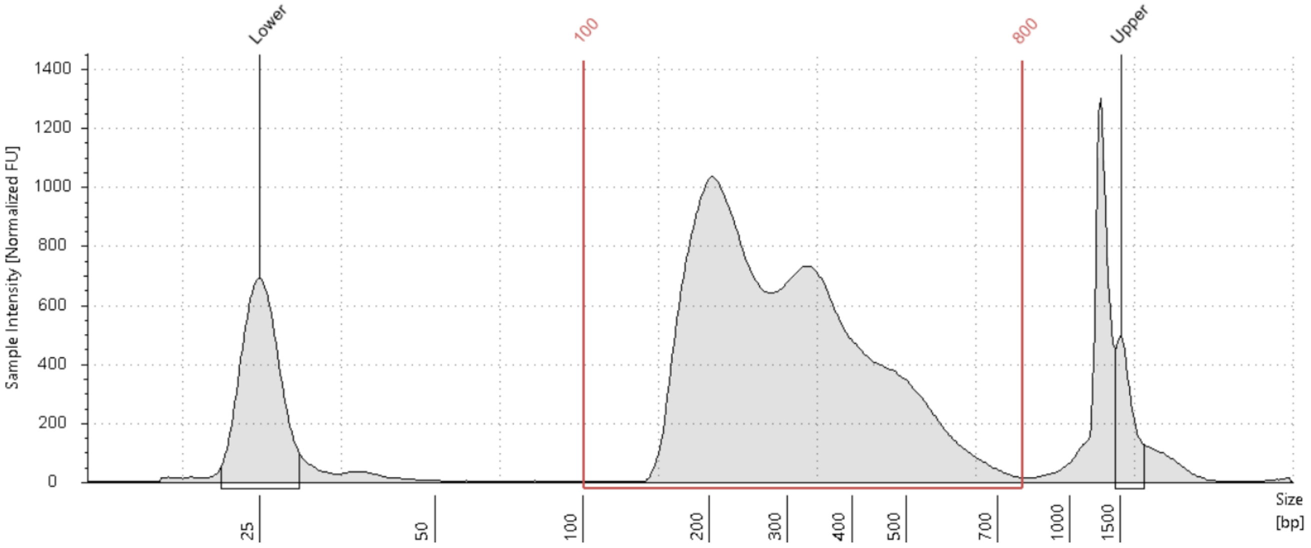

Region Table

From [bp]	To [bp]	Average Size [bp]	Conc. [pg/µl]	Region Molarity [pmol/l]	% of Total	Region Comment	Color
100	800	314	4650	26000	84.77		

© Agilent Technologies, Inc. 2021

F1: 238544

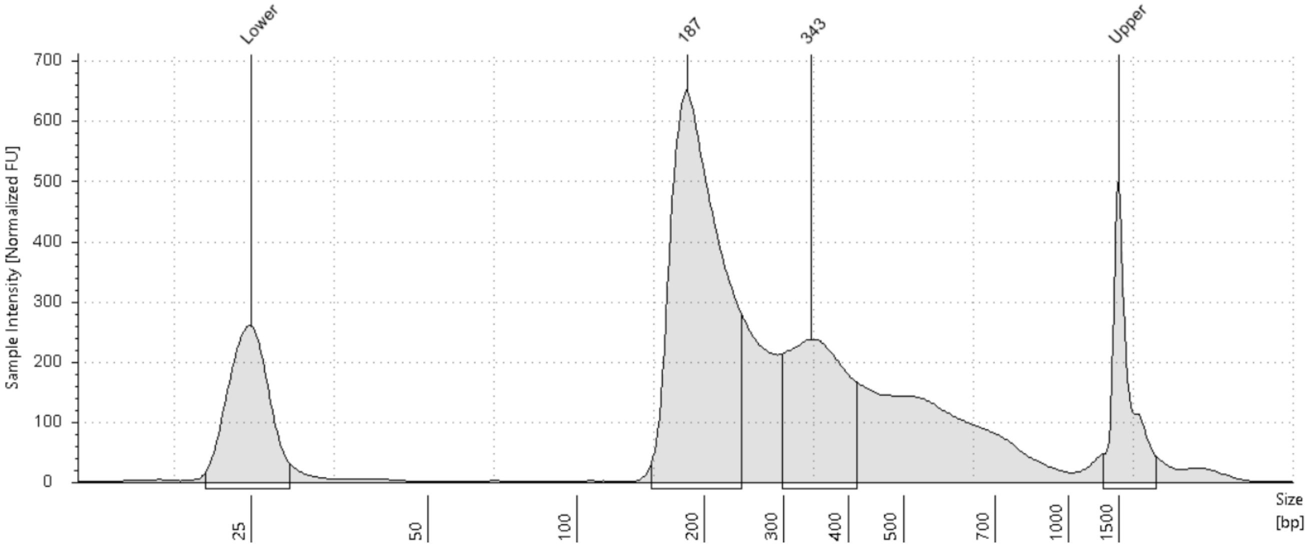

Sample Table

Well	Conc. [pg/µl]	Sample Description	Alert	Observations
F1	1490	238544	⚠	Caution! Expired ScreenTape device

Peak Table

Size [bp]	Calibrated Conc. [pg/µl]	Assigned Conc. [pg/µl]	Peak Molarity [pmol/l]	% Integrated Area	Peak Comment	Observations
25	345	-	21200	-		Lower Marker
187	1040	-	8550	69.60		
343	453	-	2030	30.40		
1500	250	250	256	-		Upper Marker

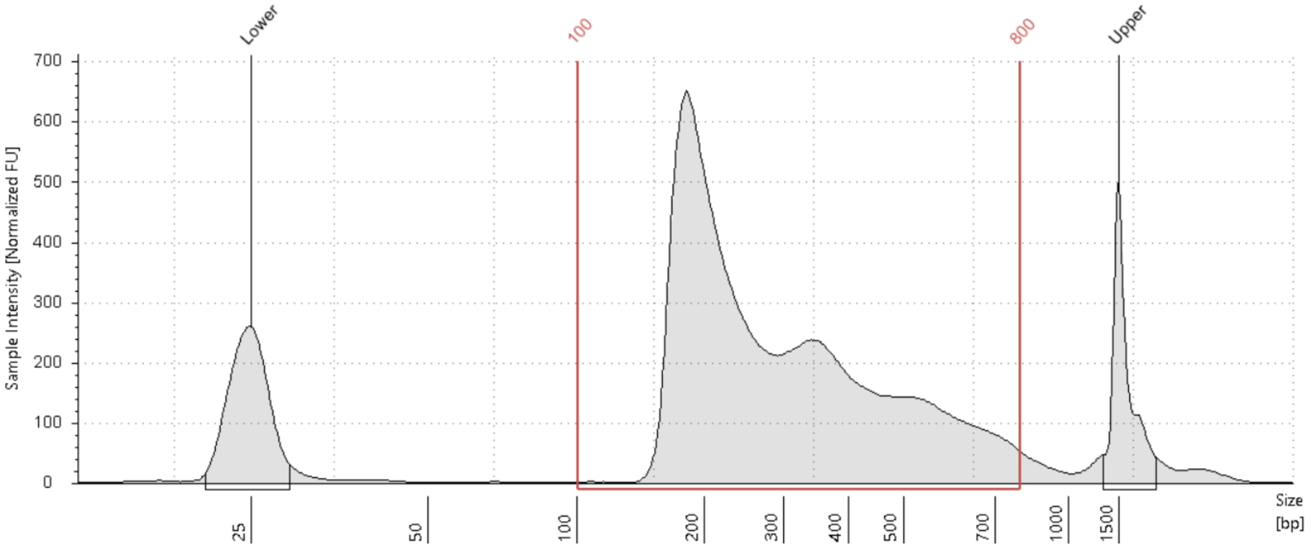

Region Table

From [bp]	To [bp]	Average Size [bp]	Conc. [pg/µl]	Region Molarity [pmol/l]	% of Total	Region Comment	Color
100	800	321	2290	13200	94.02		

 © Agilent Technologies, Inc. 2021

G1: 238545

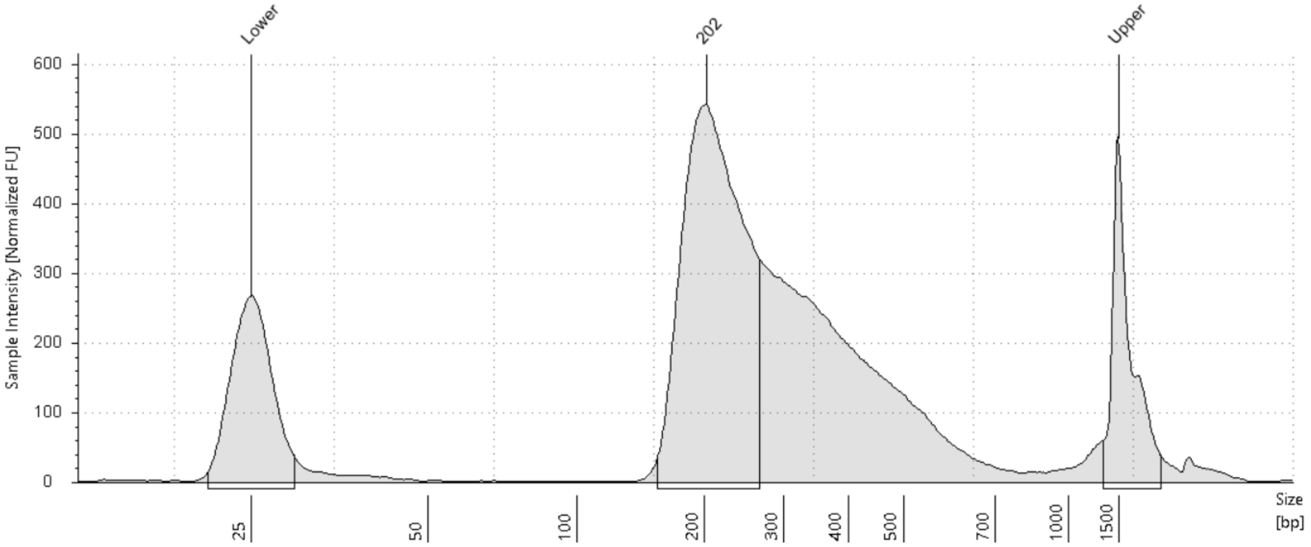

Sample Table

Well	Conc. [pg/µl]	Sample Description	Alert	Observations
G1	908	238545	⚠	Caution! Expired ScreenTape device

Peak Table

Size [bp]	Calibrated Conc. [pg/µl]	Assigned Conc. [pg/µl]	Peak Molarity [pmol/l]	% Integrated Area	Peak Comment	Observations
25	302	-	18600	-		Lower Marker
202	908	-	6910	100.00		
1500	250	250	256	-		Upper Marker

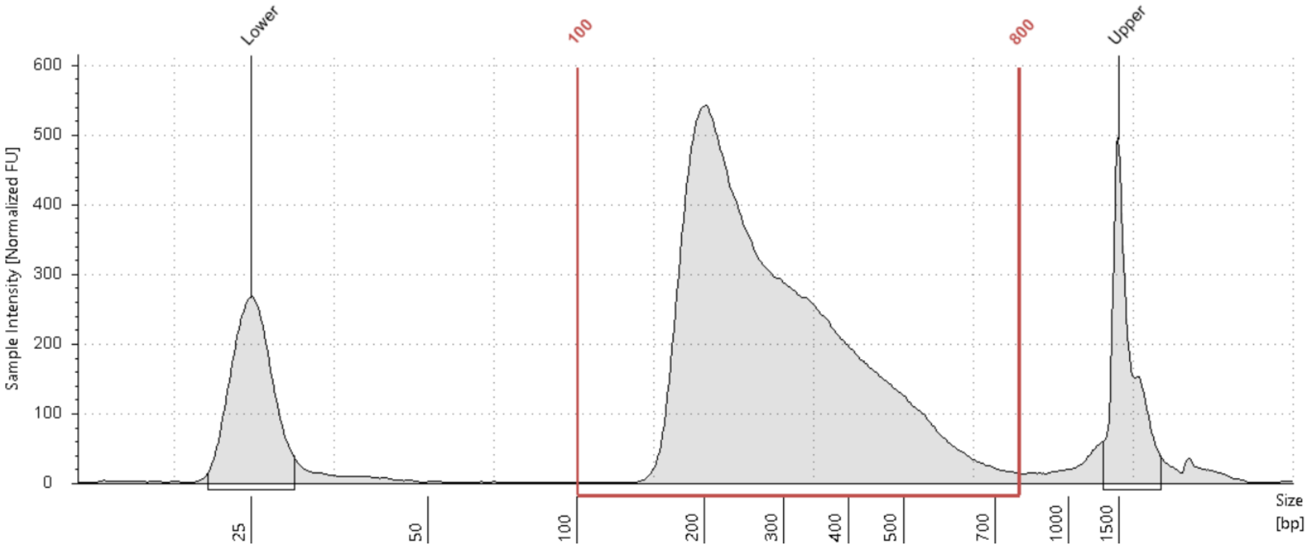

Region Table

From [bp]	To [bp]	Average Size [bp]	Conc. [pg/µl]	Region Molarity [pmol/l]	% of Total	Region Comment	Color
100	800	306	1810	10400	94.20		■

© Agilent Technologies, Inc. 2021
